# Supplementary material for: Hydroxylation of HPPD facilitates its PUB11-mediated ubiquitination and degradation in response to oxidative stress in Arabidopsis
Source: Plant Commun. 2025 Sep 8;6(11):101521. doi: 10.1016/j.xplc.2025.101521 (PMC12785156; doi:10.1016/j.xplc.2025.101521)
Supplement: Document S1. Supplemental Figures 1–17 and Supplemental Tables 1, 2, and 4 [file mmc1.pdf]

Plant Communications, Volume *6*

Supplemental information

Hydroxylation of HPPD facilitates its PUB11-mediated ubiquitination and degradation in response to oxidative stress in *Arabidopsis*

Xin-He Yu, Xun Wen, Jiangqing Dong, Ya-Fang Hu, Xin-Long Wang, Dan-Yi Zhu, Qihua Ling, Hong-Yan Lin, and Guang-Fu Yang

Supporting information

Hydroxylation of HPPD facilitates its PUB11-mediated ubiquitination and degradation in response to oxidative stress in *Arabidopsis*

Xin-He Yu[1,2]†, Xun Wen[1,2]†, Jiangqing Dong[3,4]†, Ya-Fang Hu[1,2], Xin-Long Wang[1,2], Dan-Yi Zhu[1,2], Qihua Ling[5,6]*, Hong-Yan Lin[1,2]*, Guang-Fu Yang[1,2]*

[1]State Key Laboratory of Green Pesticide, Central China Normal University, Wuhan 430079, PR China.

[2]International Joint Research Center for Intelligent Biosensor Technology and Health, Central China Normal University, Wuhan 430079, PR China.

[3]Hubei Shizhen Laboratory, Wuhan 430061, PR China.

[4]School of Basic Medical Sciences, Hubei University of Chinese Medicine, Wuhan 430065, PR China.

[5]Key Laboratory of Plant Carbon Capture, CAS Centre for Excellence in Molecular Plant Sciences, Institute of Plant Physiology and Ecology, Chinese Academy of Sciences, Shanghai, China.

[6]CAS-JIC Center of Excellence for Plant and Microbial Sciences (CEPAMS), Institute of Plant Physiology and Ecology, Chinese Academy of Sciences, Shanghai, China.

***Corresponding author. Email: qhling@cemps.ac.cn;hylin@ccnu.edu.cn; gfyang@ccnu.edu.cn.**

†These authors contributed equally to this work.

The file includes:

Figs. S1 to S17

Tables S1 to S4

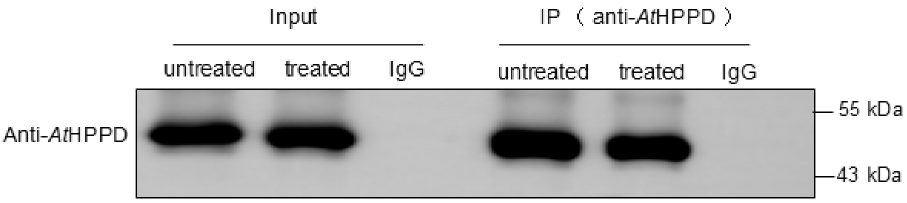

Fig. S1. *In vivo* enrichment of *At*HPPD in IP assays. Two-week-old WT plants were grown under normal growth condition, or subjected to H_2O_2 stress, with H_2O_2 treatment for 24 h. The protein extract from the plants was analyzed by IP using *At*HPPD antibody, or IgG as a negative control, and analyzed by immunoblotting using antibodies as indicated to the image.

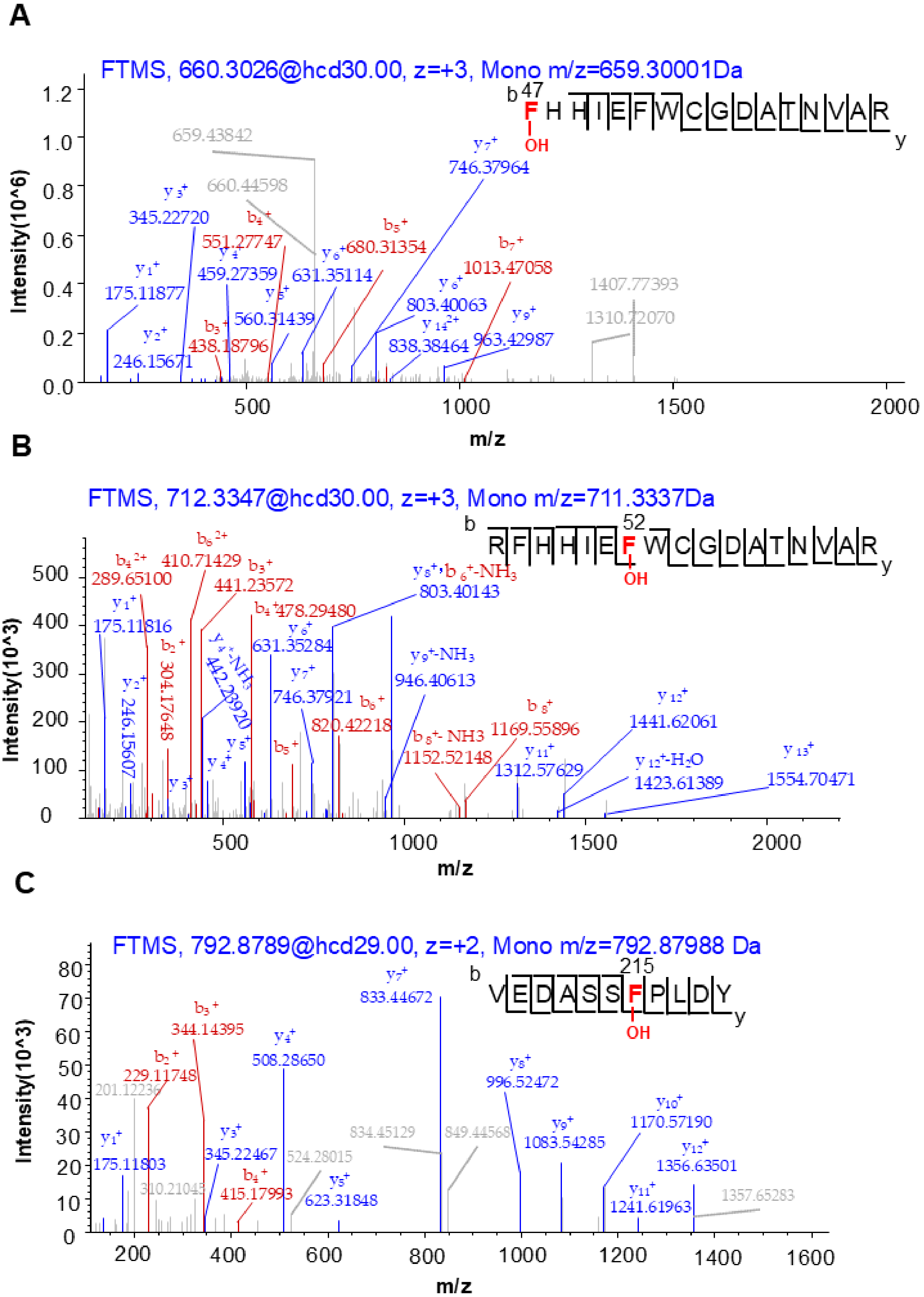

A
FTMS, 660.3026@hcd30.00, z=+3, Mono m/z=659.30001Da
b 47
F H H I E F W C G D A T N V A R
OH
y
Intensity(10^6)
m/z
659.43842
660.44598
1407.77393
1310.72070
y1+ 175.11877
y2+ 246.15671
y3+ 345.22720
b3+ 438.18796
y4+ 459.27359
b4+ 551.27747
y5+ 560.31439
y6+ 631.35114
b5+ 680.31354
y7+ 746.37964
y6+ 803.40063
y14 2+ 838.38464
y9+ 963.42987
b7+ 1013.47058
B
FTMS, 712.3347@hcd30.00, z=+3, Mono m/z=711.3337Da
b 52
R F H H I E F W C G D A T N V A R
OH
y
Intensity(10^3)
m/z
y1+ 175.11816
y2+ 246.15607
b4 2+ 289.65100
b2+ 304.17648
b6 2+ 410.71429
b3+ 441.23572
y4+-NH3 442.23920
b4+ 478.29480
y6+ 631.35284
y7+ 746.37921
y8+, b6+-NH3 803.40143
b6+ 820.42218
y9+-NH3 946.40613
b8+- NH3 1152.52148
b8+ 1169.55896
y11+ 1312.57629
y12+-H2O 1423.61389
y12+ 1441.62061
y13+ 1554.70471
C
FTMS, 792.8789@hcd29.00, z=+2, Mono m/z=792.87988 Da
b 215
V E D A S S F P L D Y
OH
y
Intensity(10^3)
m/z
y1+ 175.11803
201.12236
b2+ 229.11748
310.21045
b3+ 344.14395
y3+ 345.22467
b4+ 415.17993
y4+ 508.28650
524.28015
y5+ 623.31848
y7+ 833.44672
834.45129
849.44568
y8+ 996.52472
y9+ 1083.54285
y10+ 1170.57190
y11+ 1241.61963
y12+ 1356.63501
1357.65283

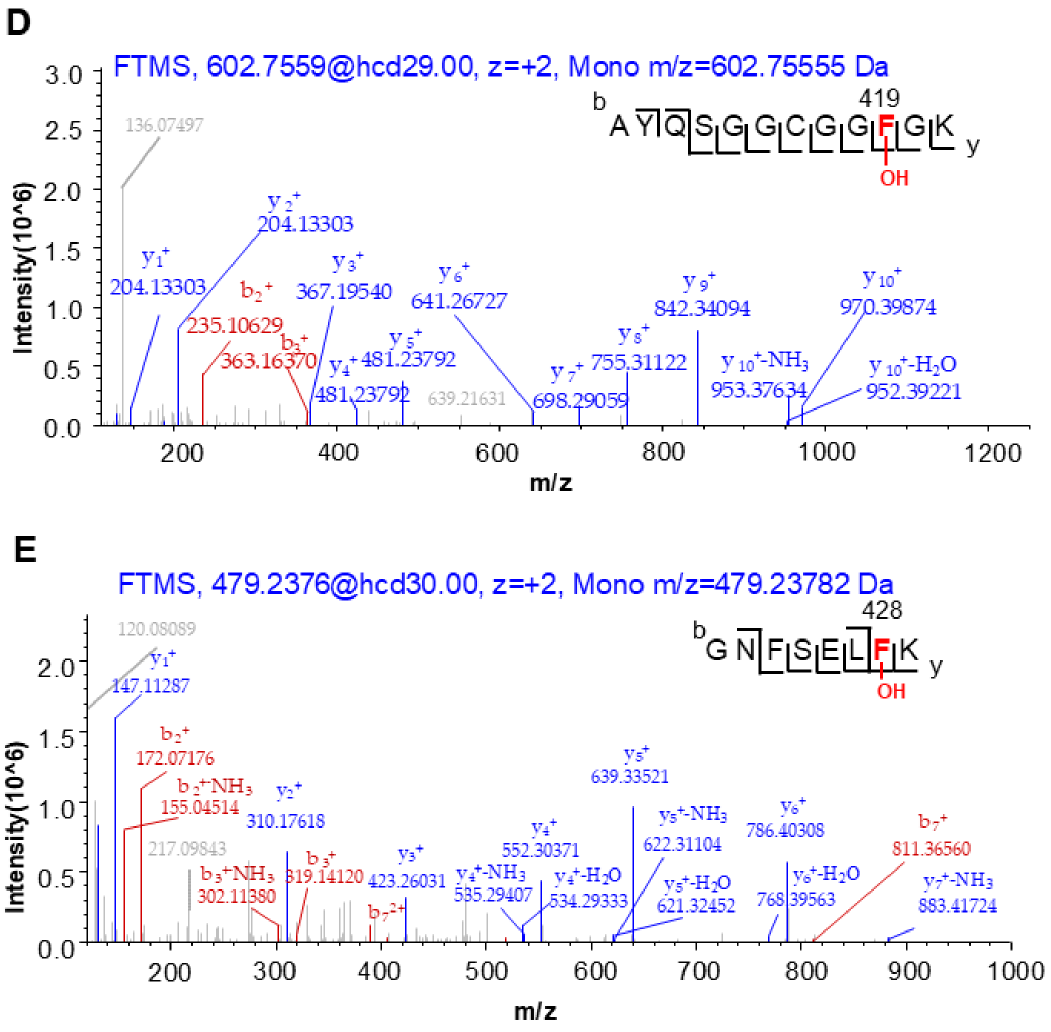

Fig. S2. Identification of HPPD modification sites *in vivo* by LC-MS/MS. (A) The product ion spectrum of the residue F47 of *At*HPPD. (B) The product ion spectrum of the residue F52 of *At*HPPD. (C) The product ion spectrum of the residue F215 of *At*HPPD. (D) The product ion spectrum of the residue F419 of *At*HPPD. (E) The product ion spectrum of the residue F428 of *At*HPPD.

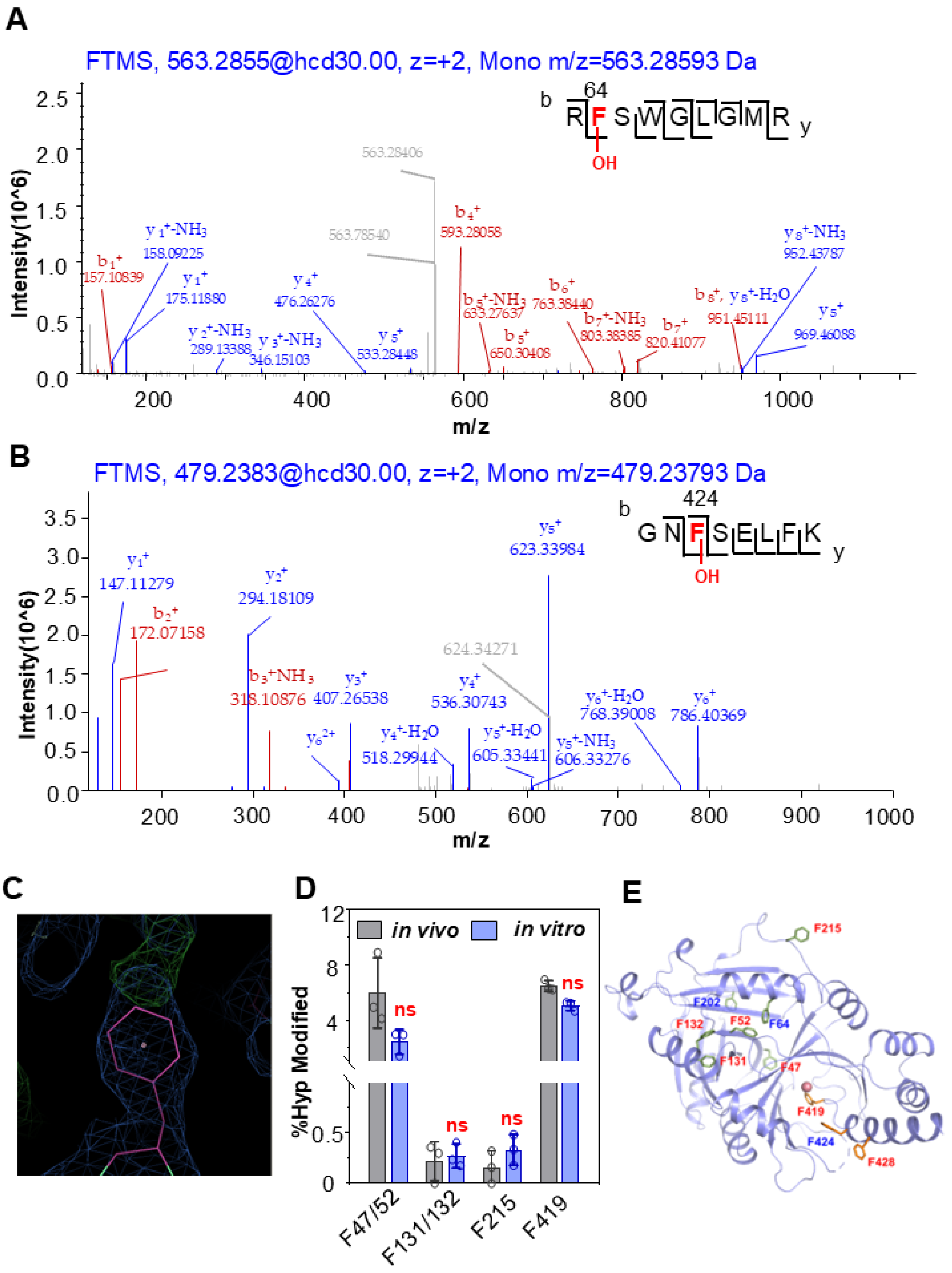

Fig. S3. Identification of HPPD modification sites *in vitro* by LC-MS/MS and crystal structures. (A) The product ion spectrum of the residue F64 of *At*HPPD. (B) The product ion spectrum of the residue F424 of *At*HPPD. (C) Density map of F132 sidechain in the *At*HPPD crystal structures showed the modification of hydroxylation. (D) Comparison of hydroxylation levels *in vitro* and *in vivo*. ns means not significant. %Hyp Modified refers to the level of hydroxylation. (E) Hydroxylation sites identified by LC-MS/MS. Red indicates mofied sites in both *in vivo* and *in vitro*;

blue indicates modified sites only *in vitro*.

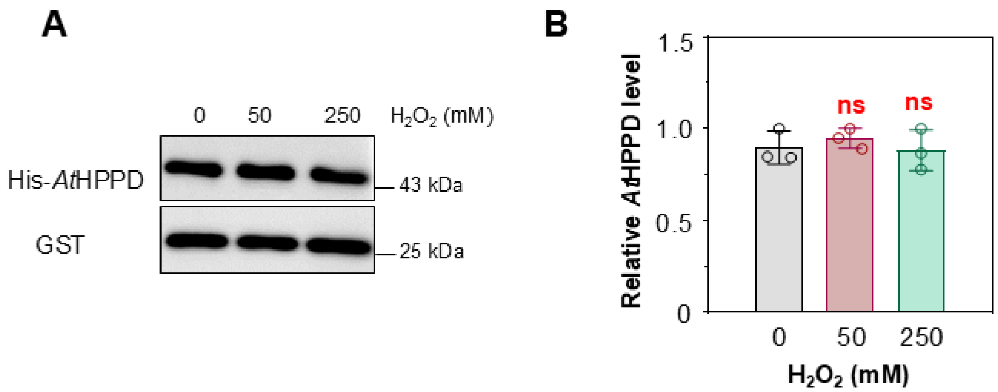

Fig. S4. H_2O_2 cannot directly degrade *At*HPPD. (A) The degradation of *At*HPPD is not directly induced in the presence of 50 mM and 250 mM H_2O_2. GST served as a loading control. (B) Relative band densities of the His-*At*HPPD protein shown in (A), as quantified using Touch view with 0 h set to 1. Data are means of three replicates, and the individual results for each replicate are shown. Significant differences compared with 0 h were determined using Student's t-test: ns means not significant.

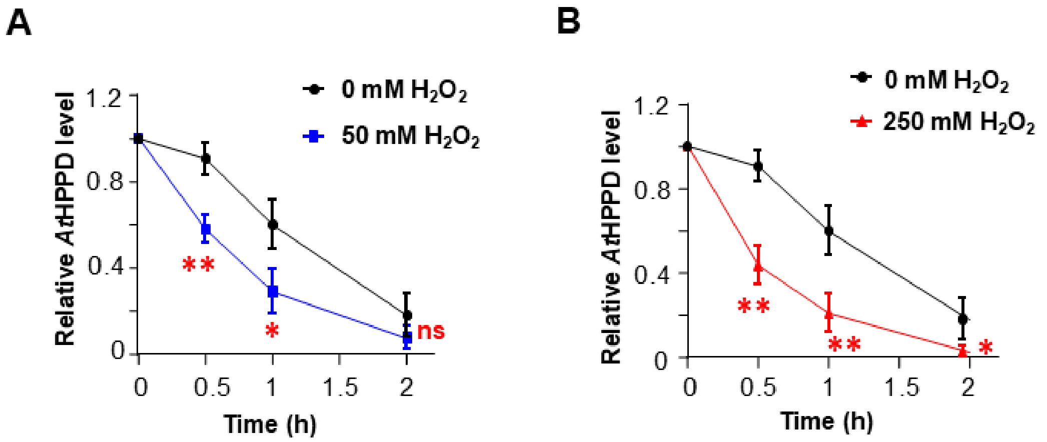

Fig. S5. The effect of H_2O_2 on the stability of *At*HPPD in an *in vitro* cell-free degradation assay. (A) Dose-response curves of the relative *At*HPPD levels at 0 mM and 50 mM H_2O_2 shown in (Fig. 2A). (B) Dose-response curves of the relative *At*HPPD levels at 0 mM and 250 mM H_2O_2 shown in (Fig. 2A). Data are means of three replicates, and the individual results for each replicate are shown. * means $P < 0.05$, ** means $P < 0.01$, ns means not significant.

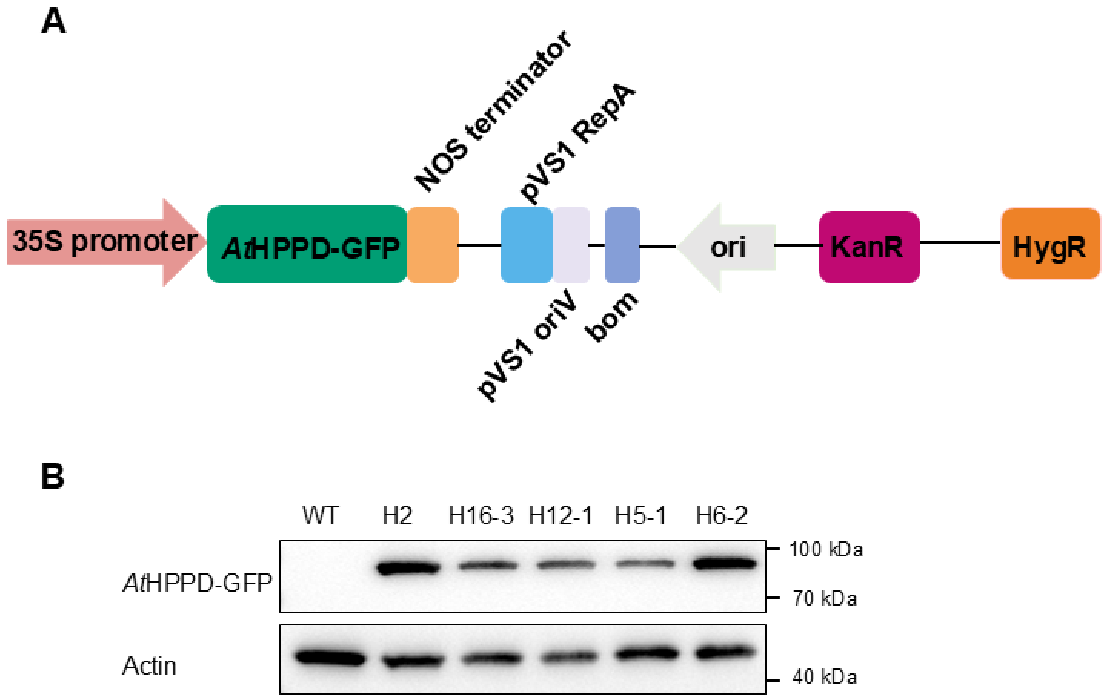

Fig. S6. Expression level of transgenic lines expressing *At*HPPD-GFP. (A) The pBWA(V)HS vector for expression of *At*HPPD in *Arabidopsis thaliana*. KanR and HygR, kanamycin and hygromycin resistance genes. (B) Expression profiles determined by immunoblotting analysis. Actin served as a loading control.

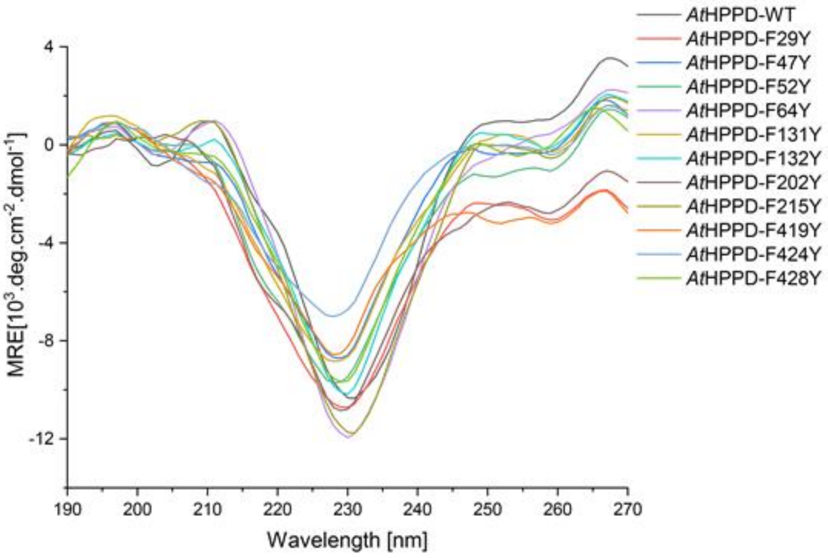

Fig. S7. CD spectra of *At*HPPD and its mutants.

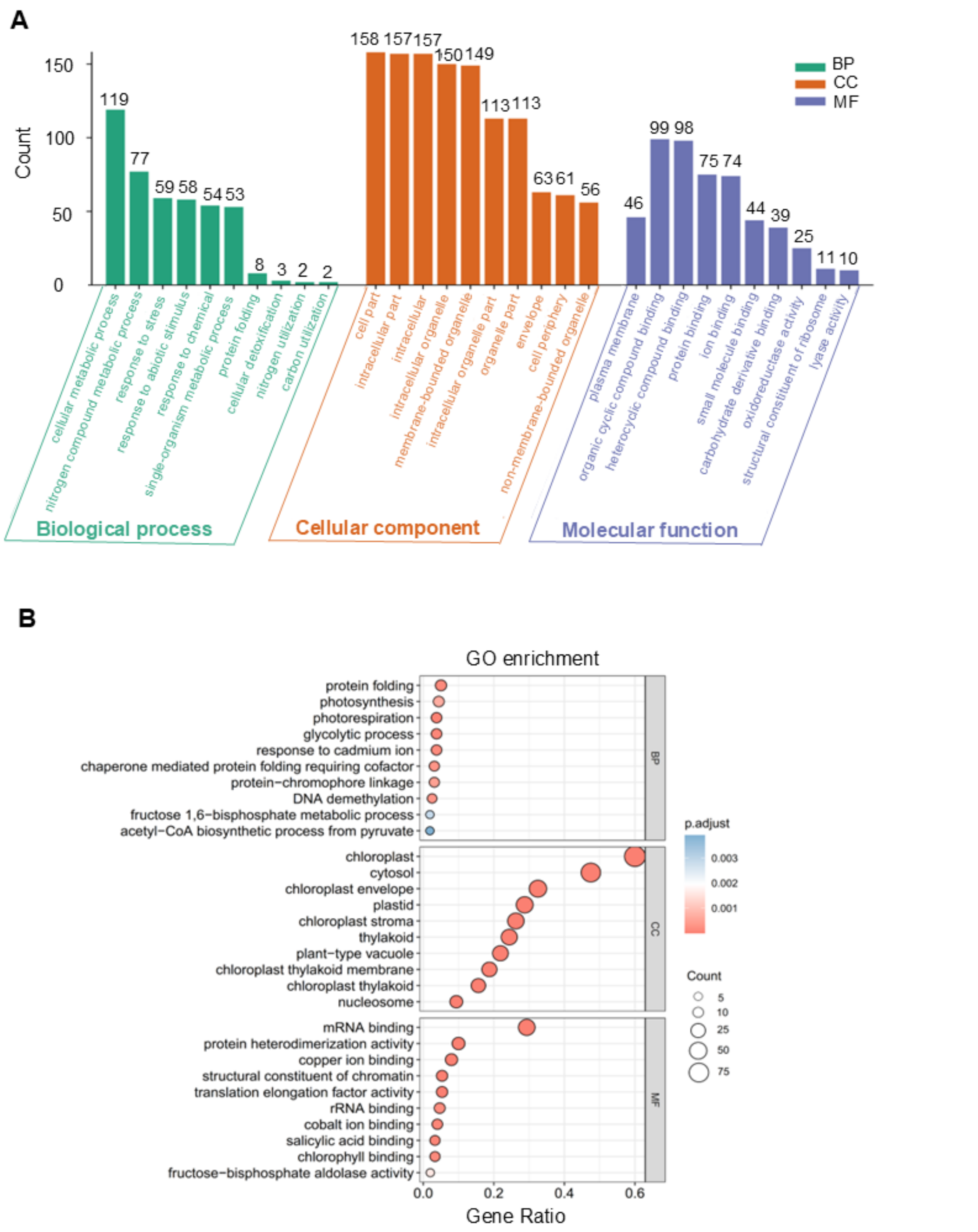

Fig. S8. GO classification and enrichment analysis of the identified the interaction proteins of *At*HPPD. (A) Functional annotation of the interaction proteins of *At*HPPD based on biological process, cellular component and molecular function, which were significantly in accordance with hyper p value which is < 0.05. BP means biological process, CC means cellular component, MF means molecular function. (B) GO enrichment analysis of the interaction proteins of *At*HPPD. A higher gene ratio indicates a greater level of enrichment. The size of the circles in the figure represents the number of proteins associated with each GO category, with larger circles indicating a higher number of associated proteins.

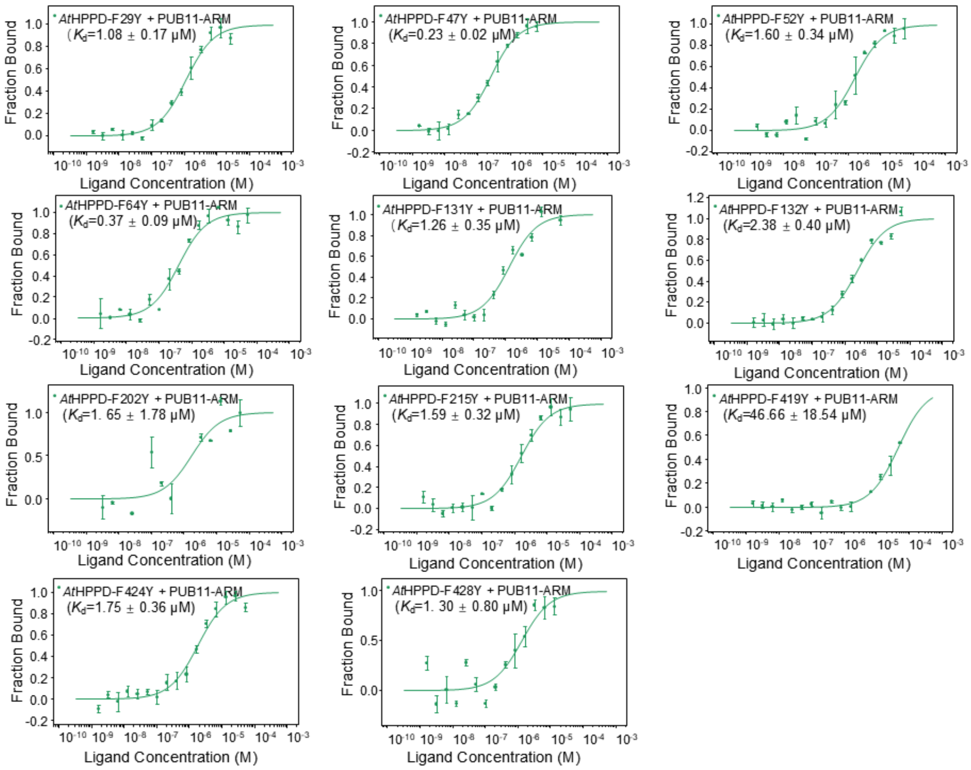

Fig. S9. MST-based interaction analysis between hydroxylated *At*HPPD mutants and PUB11.

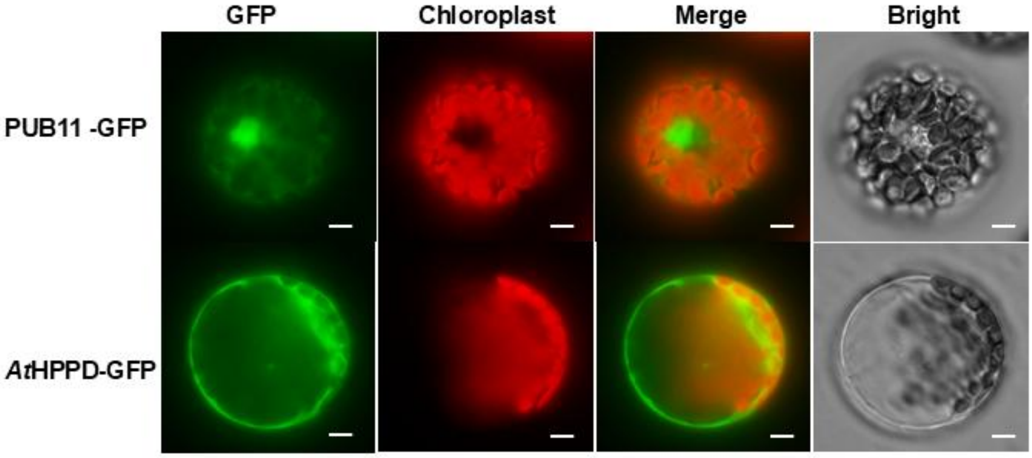

Fig. S10. Subcellular localization of PUB11 and *At*HPPD. PUB11-GFP and *At*HPPD-GFP constructs were respectively transformed into *Arabidopsis protoplasts*. Bars = 10 μm.

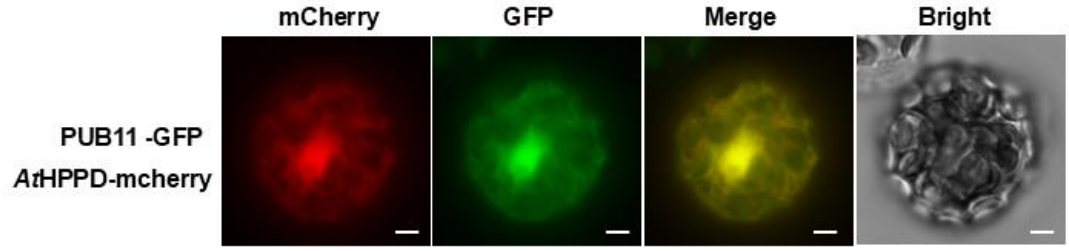

Fig. S11. Co-localization of PUB11 and *At*HPPD. PUB11-GFP and *At*HPPD-mcherry constructs were co-transformed into *Arabidopsis* protoplasts. Bars = 10 µm.

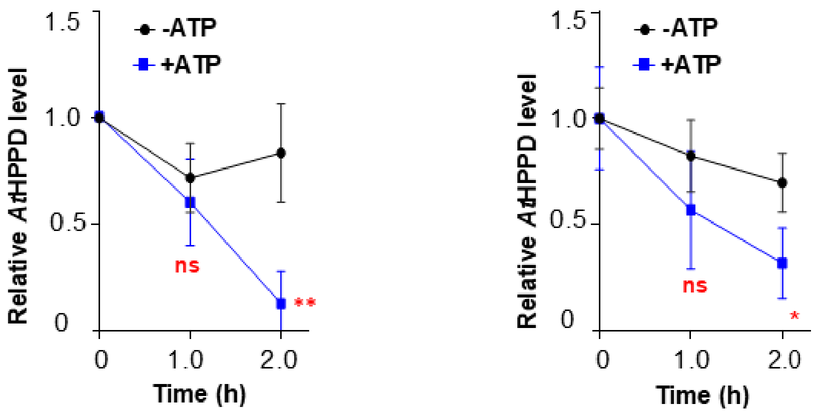

Fig. S12. ATP-dependent enhancement of *At*HPPD degradation. Dose–response curves of relative band intensity of the *At*HPPD-GFP protein in *At*HPPD-GFP-OE (H2 and H6-2) plants shown in (Fig. 4A), as quantified using Touch view with 0 h set to 1 Data are means of three replicates, and the individual results for each replicate are shown. The abundance of *At*HPPD at the 0 min (-ATP, +ATP) was set to 1, respectively. Significant differences compared with 0 h were determined using Student's t-test: * means P < 0.05, ** means P < 0.01, ns means not significant.

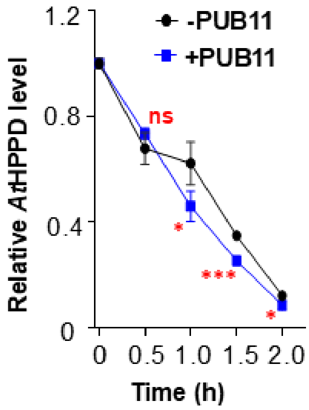

Fig. S13. PUB11 enhances *At*HPPD degradation *in vitro*. Dose–response curves of relative band intensity of the *At*HPPD protein shown in (Fig.4D), as quantified using touch view with 0 h set to 1. Data are means of three replicates, and the individual results for each replicate are shown. Significant differences compared with 0 h were determined using Student's t-test: * means $P < 0.05$, *** means $P < 0.001$, ns means not significant.

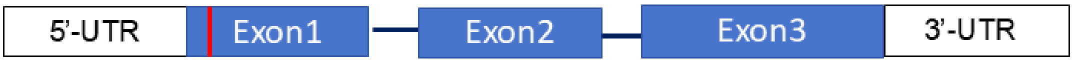

Target: CCGGAG-GAATCGTCTCACCGGCC:

WT: GCGACGGCGTAATGGCCGGAGGAATCGTCTCACCGGCCTCTCTA
pub11(#1): GCGACGGCGTAATGGCCGGAGTGAATCGTCTCACCGGCCTCTCTA (insert 1bp)
pub11(#2): GCGACGGCGTAATGGCCGGAG-GAATCGTCTCACCGGCCTCTCTA (deletion 1bp)

Fig. S14. Identification of *pub11* for the creation of CRISPR/Cas9 knockout lines.

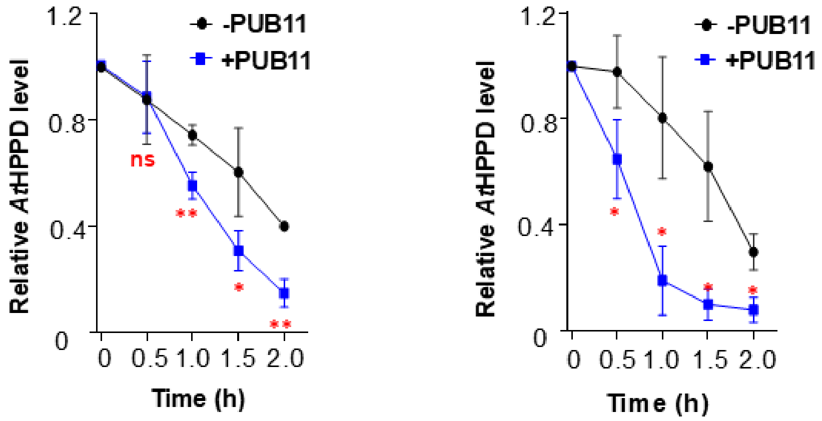

Fig. S15. Cell-free degradation of His-*At*HPPD in *pub11* extracts with or without recombinant PUB11 protein. Dose–response curves of relative band intensity of the *At*HPPD protein shown in (Fig.4E), as quantified using touch view with 0 h set to 1. Data are means of three replicates, and the individual results for each replicate are shown. Significant differences compared with 0 h were determined using Student's t-test: * means P < 0.05, ** means P < 0.01, ns means not significant.

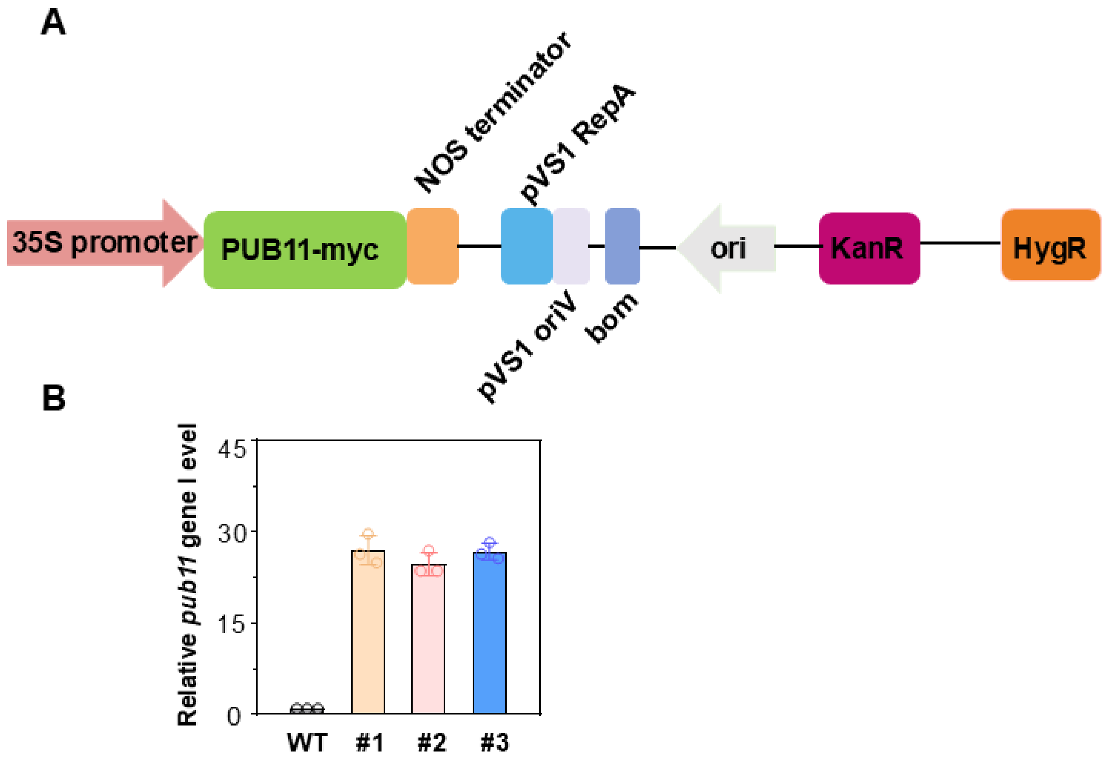

Fig. S16. Expression level of transgenic lines and wild-type plants. (A). The pCAMBIA 3300 vector for expression of PUB11-OE in *Arabidopsis thaliana*. KanR and HygR, kanamycin and hygromycin resistance genes. (B). *Arabidopsis thaliana* RT-PCR results for transgenic gene. WT, wild type plants; #1~#3, PUB11 overexpressing plants.

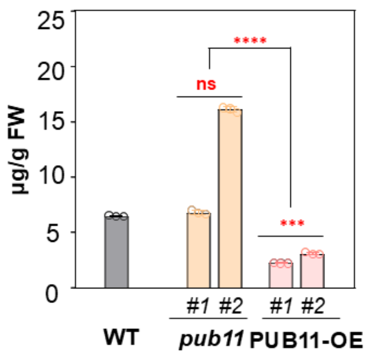

Fig. S17. Tocopherol content determination. *** means $P < 0.001$, **** means $P < 0.0001$, ns means not significant. Error bars indicate ±s.d.

Table S1 *At*HPPD mutants exhibited no detectable hydroxylation modifications

Mutants	Annotated sequence	RT (min)	Hydroxylation sequence
*At*HPPD-F29A	GASKFVR	38.3912	Not identified
*At*HPPD-F47A	RAHHIEFWCGDATNVATNVA	32.5861	Not identified
*At*HPPD-F52A	FHHIEAWCGDATNVATNVA	34.1440	Not identified
*At*HPPD-F64A	RASWGLGMR	20.9285	Not identified
*At*HPPD-F131A	SAFSSHGLGVR	20.1026	Not identified
*At*HPPD-F132A	SFASSHGLGVR	15.8525	Not identified
*At*HPPD-F202A	AEDTEKSEALPGFER	21.2350	Not identified
*At*HPPD-F215A	VEDASSAPLDYGIR	31.4586	Not identified
*At*HPPD-F419A	AYQSGGCGGAGK	8.3512	Not identified
*At*HPPD-F424A	GNASELFK	37.0387	Not identified
*At*HPPD-F428A	KGNFSELAK	12.8025	Not identified

Table S2 Comparison of apparent kinetic parameters for reaction of the *At*HPPD with H_2O_2. Each experiment was carried out in triplicate.

The concentration of H_2O_2(mM)	K_m(μM)	k_{cat}(s^{-1})	k_{cat}/K_m($s^{-1}μM^{-1}$)
0	2.061 ± 0.069	0.200 ± 0.010	0.097
50	2.699 ± 0.071	0.070 ± 0.004	0.026
250	12.354 ± 0.610	0.076 ± 0.001	0.006

Table S4 Oligonucleotide primers used in the study

Primer	Sequence（5' to 3'）
GFP-*At*HPPD-F	GAGCATATACGCCCGGAGTC
GFP-*At*HPPD-R	CAAGACCTGCCTGAAACCGA
pub11-F	TGCGATACCTGCCTTAGTT
pub11-R	GGCTCTGACTGCTCTACCTTT
actin-F	TCCCGCTATGTATGTCGC
actin-R	GCTGGTCTTTGAGGTTTCC
