# Supplementary material for: Hydroxylation of HPPD facilitates its PUB11-mediated ubiquitination and degradation in response to oxidative stress in Arabidopsis
Source: Plant Commun. 2025 Sep 8;6(11):101521. doi: 10.1016/j.xplc.2025.101521 (PMC12785156; doi:10.1016/j.xplc.2025.101521)
Supplement: Supplemental Table 3. AtHPPD-GFP–interacting proteins identified by IP–LC–MS/MS [file mmc2.pdf]

Table S3 The interaction proteins of *At*HPPD-GFP in IP-LC-MS/MS

Accession	Description	Coverage [%]	Peptides	PSMs
P93836	4-hydroxyphenylpyruvate dioxygenase OS=Arabidopsis thaliana OX=3702 GN=HPD PE=1 SV=2	62	23	84
A0A178WCR1	RING-type E3 ubiquitin transferase OS=Arabidopsis thaliana OX=3702 GN=AXX17_At1g24190 PE=4 SV=1	1	1	1
A0A075M556	Ribulose bisphosphate carboxylase large chain (Fragment) OS=Arabidopsis thaliana OX=3702 GN=rbcL PE=3 SV=1	45	22	28
A0A178UCH2	TPT OS=Arabidopsis thaliana OX=3702 GN=AXX17_At5g44540 PE=4 SV=1	3	1	1
A0A178UDC6	UBQ9 OS=Arabidopsis thaliana OX=3702 GN=AXX17_At5g34780 PE=4 SV=1	27	4	6
A0A178UDD3	Histone H2B OS=Arabidopsis thaliana OX=3702 GN=AXX17_At5g22390 PE=3 SV=1	16	2	2
A0A178UE59	DDE2 OS=Arabidopsis thaliana OX=3702 GN=AXX17_At5g40520 PE=4 SV=1	2	1	1
A0A178UEX9	CRR1 OS=Arabidopsis thaliana OX=3702 GN=At5g52100 PE=3 SV=1	4	1	1
A0A178UMW0	Malate dehydrogenase OS=Arabidopsis thaliana OX=3702 GN=AXX17_At5g09190 PE=3 SV=1	6	2	2
A0A178UUR9	VDAC2 OS=Arabidopsis thaliana OX=3702 GN=AXX17_At5g67610 PE=3 SV=1	3	1	1
A0A178UVK9	ELI3-2 OS=Arabidopsis thaliana OX=3702 GN=AXX17_At4g43300 PE=3 SV=1	3	1	1
A0A178V017	(thale cress) hypothetical protein OS=Arabidopsis thaliana OX=3702 GN=At4g32260 PE=1 SV=1	4	1	1
A0A178V098	(thale cress) hypothetical protein OS=Arabidopsis thaliana OX=3702 GN=AXX17_At4g45110 PE=4 SV=1	10	2	2
A0A178V385	Fructose-bisphosphate aldolase OS=Arabidopsis thaliana OX=3702 GN=AXX17_At4g30610 PE=3 SV=1	15	5	5
A0A178V984	(thale cress) hypothetical protein OS=Arabidopsis thaliana OX=3702 GN=AXX17_At3g57810 PE=4 SV=1	14	3	4
A0A178V9A9	(thale cress) hypothetical protein OS=Arabidopsis thaliana OX=3702 GN=AXX17_At3g01260 PE=3 SV=1	4	2	2
A0A178VBH7	(thale cress) hypothetical protein OS=Arabidopsis thaliana OX=3702 GN=AXX17_At3g05130 PE=3 SV=1	10	1	1
A0A178VCC0	HSP70 OS=Arabidopsis thaliana OX=3702 GN=AXX17_At3g12610 PE=3 SV=1	6	4	4

A0A178VDZ1	PTAC16 OS=Arabidopsis thaliana OX=3702 GN=AXX17_At3g40690 PE=4 SV=1	6	3	3
A0A178VE16	(thale cress) hypothetical protein OS=Arabidopsis thaliana OX=3702 GN=AXX17_At3g00320 PE=3 SV=1	5	1	1
A0A178VFM4	Chlorophyll a-b binding protein, chloroplastic OS=Arabidopsis thaliana OX=3702 GN=AXX17_At3g08510 PE=3 SV=1	7	3	3
A0A178VK37	UDP-arabinopyranose mutase OS=Arabidopsis thaliana OX=3702 GN=AXX17_At3g01420 PE=3 SV=1	3	1	1
A0A178VKH7	ALDH2B4 OS=Arabidopsis thaliana OX=3702 GN=At3g48000 PE=3 SV=1	2	1	1
A0A178VL51	Uncharacterized protein OS=Arabidopsis thaliana OX=3702 GN=AXX17_At3g42150 PE=4 SV=1	7	1	1
A0A178VLH1	Non-specific lipid-transfer protein OS=Arabidopsis thaliana OX=3702 GN=AXX17_At3g45940 PE=3 SV=1	25	4	4
A0A178VPL4	Homoserine kinase OS=Arabidopsis thaliana OX=3702 GN=AXX17_At2g12440 PE=3 SV=1	8	1	1
A0A178VRN7	Fructose-bisphosphate aldolase OS=Arabidopsis thaliana OX=3702 GN=AXX17_At2g33200 PE=3 SV=1	11	4	4
A0A178VRV1	Superoxide dismutase [Cu-Zn] OS=Arabidopsis thaliana OX=3702 GN=AXX17_At2g24200 PE=3 SV=1	10	2	2
A0A178VU15	Serine/threonine-protein kinase RIO1 OS=Arabidopsis thaliana OX=3702 GN=AXX17_At2g20700 PE=3 SV=1	2	1	1
A0A178VW63	(thale cress) hypothetical protein OS=Arabidopsis thaliana OX=3702 GN=At2g39730 PE=4 SV=1	16	7	7
A0A178W1G4	RING-type domain-containing protein OS=Arabidopsis thaliana OX=3702 GN=AXX17_At2g31900 PE=4 SV=1	1	1	1
A0A178W2K5	(thale cress) hypothetical protein OS=Arabidopsis thaliana OX=3702 GN=AXX17_At2g28120 PE=4 SV=1	1	1	1
A0A178W987	Carbonic anhydrase OS=Arabidopsis thaliana OX=3702 GN=AXX17_At1g64540 PE=3 SV=1	3	1	1
A0A178WBG3	Prohibitin OS=Arabidopsis thaliana OX=3702 GN=AXX17_At1g03130 PE=3 SV=1	4	1	1
A0A178WCD3	RING-type E3 ubiquitin transferase OS=Arabidopsis thaliana OX=3702 GN=AXX17_At1g43980 PE=4 SV=1	2	1	1
A0A178WDB6	Uncharacterized protein OS=Arabidopsis thaliana OX=3702 GN=AXX17_At1g20520 PE=4 SV=1	14	2	2
A0A178WF48	KV-BETA1 OS=Arabidopsis thaliana OX=3702 GN=AXX17_At1g04030 PE=3 SV=1	2	1	1
A0A178WHK4	Ubiquitin OS=Arabidopsis thaliana OX=3702 GN=AXX17_At1g24630 PE=3 SV=1	24	3	4

A0A178WI60	Signal recognition particle subunit SRP72 OS=Arabidopsis thaliana OX=3702 GN=At1g67680 PE=3 SV=1	2	1	1
A0A178WJ12	TLL1 OS=Arabidopsis thaliana OX=3702 GN=AXX17_At1g40860 PE=4 SV=1	2	1	1
A0A178WKB6	Glyceraldehyde-3-phosphate dehydrogenase OS=Arabidopsis thaliana OX=3702 GN=AXX17_At1g39030 PE=3 SV=1	13	3	3
A0A1B1W4T6	Photosystem I P700 chlorophyll a apoprotein A1 OS=Arabidopsis thaliana OX=3702 GN=psaA PE=3 SV=1	2	2	2
A0A1B1W4V8	Cytochrome f OS=Arabidopsis thaliana OX=3702 GN=petA PE=3 SV=1	5	1	1
A0A1B1W4Y0	50S ribosomal protein L14, chloroplastic OS=Arabidopsis thaliana OX=3702 GN=rpl14 PE=3 SV=1	8	1	1
A0A1P8AXC1	FtsH extracellular protease family OS=Arabidopsis thaliana OX=3702 GN=VAR2 PE=1 SV=1	3	2	2
A0A1P8AYP6	Transmembrane protein OS=Arabidopsis thaliana OX=3702 GN=At2g05310 PE=4 SV=1	12	1	1
A0A1P8B9K9	ATP:AMP phosphotransferase OS=Arabidopsis thaliana OX=3702 GN=AMK2 PE=1 SV=1	3	1	1
A0A1P8BD41	NADH-ubiquinone oxidoreductase-like protein OS=Arabidopsis thaliana OX=3702 GN=MXC20.6 PE=1 SV=1	8	1	1
A0A1P8BGK1	Transmembrane protein OS=Arabidopsis thaliana OX=3702 GN=MNA5.17 PE=1 SV=1	1	1	1
A0A2P2CLF9	ATP synthase subunit alpha OS=Arabidopsis thaliana OX=3702 GN=atp1 PE=3 SV=1	4	2	2
A0A384KFF7	Uncharacterized protein OS=Arabidopsis thaliana OX=3702 GN=AXX17_At2g40490 PE=3 SV=1	8	2	2
A0A384KYV2	(thale cress) hypothetical protein OS=Arabidopsis thaliana OX=3702 GN=AXX17_At3g08150 PE=3 SV=1	14	6	6
A0A5S9UF63	Uncharacterized protein OS=Arabidopsis thaliana OX=3702 GN=C24_LOCUS1528 PE=3 SV=1	1	1	1
A0A5S9WML4	Dihydrolipoyl dehydrogenase OS=Arabidopsis thaliana OX=3702 GN=C24_LOCUS3799 PE=3 SV=1	4	2	2
A0A5S9WTW9	Beta-galactosidase OS=Arabidopsis thaliana OX=3702 GN=C24_LOCUS6220 PE=3 SV=1	1	1	1
A0A5S9WUL8	Histone domain-containing protein OS=Arabidopsis thaliana OX=3702 GN=AN1_LOCUS6600 PE=3 SV=1	7	1	1
A0A5S9X1Y3	Uncharacterized protein OS=Arabidopsis thaliana OX=3702 GN=AN1_LOCUS9172 PE=4 SV=1	27	2	2
A0A5S9X4Q1	(thale cress) hypothetical protein OS=Arabidopsis thaliana OX=3702 GN=AN1_LOCUS10176 PE=3 SV=1	4	1	1
A0A5S9X597	Uncharacterized protein OS=Arabidopsis thaliana OX=3702 GN=AN1_LOCUS10181 PE=4 SV=1	3	1	1

A0A5S9XC59	Dihydrolipoyllysine-residue acetyltransferase OS=Arabidopsis thaliana OX=3702 GN=C24_LOCUS12584 PE=3 SV=1	2	1	1
A0A5S9XCL6	FMN_dh domain-containing protein OS=Arabidopsis thaliana OX=3702 GN=C24_LOCUS12641 PE=4 SV=1	5	2	2
A0A5S9XCY7	Uncharacterized protein OS=Arabidopsis thaliana OX=3702 GN=C24_LOCUS12452 PE=3 SV=1	2	1	1
A0A5S9XD25	Uncharacterized protein OS=Arabidopsis thaliana OX=3702 GN=C24_LOCUS12865 PE=4 SV=1	3	2	2
A0A5S9XP40	Epimerase domain-containing protein OS=Arabidopsis thaliana OX=3702 GN=C24_LOCUS16576 PE=4 SV=1	2	1	1
A0A5S9XRI1	Uncharacterized protein OS=Arabidopsis thaliana OX=3702 GN=C24_LOCUS17698 PE=4 SV=1	4	1	1
A0A5S9XU45	Uncharacterized protein OS=Arabidopsis thaliana OX=3702 GN=C24_LOCUS18615 PE=4 SV=1	5	1	1
A0A5S9XV13	Gamma-ECS OS=Arabidopsis thaliana OX=3702 GN=C24_LOCUS18941 PE=3 SV=1	2	1	1
A0A5S9Y9F6	14_3_3 domain-containing protein OS=Arabidopsis thaliana OX=3702 GN=C24_LOCUS23841 PE=3 SV=1	7	2	2
A0A5S9YCV0	Uncharacterized protein OS=Arabidopsis thaliana OX=3702 GN=C24_LOCUS25108 PE=4 SV=1	2	1	1
A0A5S9YDU6	ATG16 domain-containing protein OS=Arabidopsis thaliana OX=3702 GN=C24_LOCUS25106 PE=4 SV=1	2	1	1
A0A5S9YFS8	Uncharacterized protein OS=Arabidopsis thaliana OX=3702 GN=C24_LOCUS26165 PE=3 SV=1	8	3	3
A0A654ECD8	Uncharacterized protein OS=Arabidopsis thaliana OX=3702 GN=AN1_LOCUS2420 PE=3 SV=1	15	2	2
A0A654EIJ1	Uncharacterized protein OS=Arabidopsis thaliana OX=3702 GN=AN1_LOCUS4628 PE=4 SV=1	17	2	3
A0A654ERW1	Uncharacterized protein OS=Arabidopsis thaliana OX=3702 GN=AN1_LOCUS5732 PE=4 SV=1	1	1	1
A0A654ETR6	(thale cress) hypothetical protein OS=Arabidopsis thaliana OX=3702 GN=AN1_LOCUS7603 PE=3 SV=1	5	2	2
A0A654F693	Ubiquitinyl hydrolase 1 OS=Arabidopsis thaliana OX=3702 GN=AN1_LOCUS12515 PE=3 SV=1	1	1	1
A0A654F6Z0	Uncharacterized protein OS=Arabidopsis thaliana OX=3702 GN=AN1_LOCUS12743 PE=3 SV=1	2	1	1
A0A654F735	Pectinesterase OS=Arabidopsis thaliana OX=3702 GN=AN1_LOCUS12796 PE=3 SV=1	2	1	1
A0A654FDA0	PDZ domain-containing protein OS=Arabidopsis thaliana OX=3702 GN=AN1_LOCUS14299 PE=3 SV=1	3	1	1
A0A654FLE0	Serine/threonine-protein phosphatase OS=Arabidopsis thaliana OX=3702 GN=AN1_LOCUS17107 PE=3 SV=1	1	1	1

A0A654GCG4	Non-specific lipid-transfer protein OS=Arabidopsis thaliana OX=3702 GN=AN1_LOCUS26232 PE=3 SV=1	12	1	1
A5Y7B8	Nucleoside diphosphate kinase (Fragment) OS=Arabidopsis thaliana OX=3702 GN=NDPK2 PE=3 SV=1	4	1	1
A8MQY4	Carbonic anhydrase OS=Arabidopsis thaliana OX=3702 GN=CA2 PE=1 SV=1	22	5	7
A8MRZ7	Eukaryotic translation initiation factor 4A1 OS=Arabidopsis thaliana OX=3702 GN=EIF4A1 PE=1 SV=1	3	1	1
B3LF87	At1g30230 OS=Arabidopsis thaliana OX=3702 PE=2 SV=1	3	1	1
B9DFU4	Ferredoxin--NADP(+) reductase (Fragment) OS=Arabidopsis thaliana OX=3702 GN=At1g20020 PE=2 SV=1	9	2	2
B9DGD1	Glutamine synthetase OS=Arabidopsis thaliana OX=3702 GN=At5g35630 PE=2 SV=1	3	1	1
B9DHA3	AT3G18780 protein OS=Arabidopsis thaliana OX=3702 GN=At3g18780 PE=2 SV=1	7	3	3
B9DHY6	V-ATPase 69 kDa subunit (Fragment) OS=Arabidopsis thaliana OX=3702 GN=At1g78900 PE=2 SV=1	11	4	4
C0LGR3	LRR receptor-like serine/threonine-protein kinase RGI3 OS=Arabidopsis thaliana OX=3702 GN=RGI3 PE=1 SV=1	4	1	2
C0Z2I5	AT3G08580 protein OS=Arabidopsis thaliana OX=3702 GN=At3g08580 PE=2 SV=1	5	1	1
C0Z361	Chaperonin 60 subunit beta 3, chloroplastic OS=Arabidopsis thaliana OX=3702 GN=CPN60B3 PE=1 SV=1	4	2	2
C0Z3L0	AT1G05360 protein OS=Arabidopsis thaliana OX=3702 GN=KMS2 PE=1 SV=1	2	1	1
F4HNZ6	Glyceraldehyde 3-phosphate dehydrogenase A subunit 2 OS=Arabidopsis thaliana OX=3702 GN=GAPA-2 PE=1 SV=1	18	5	5
F4HS63	p-loop containing nucleoside triphosphate hydrolases superfamily protein OS=Arabidopsis thaliana OX=3702 GN=At1g80380 PE=1 SV=1	4	2	2
F4I0N7	GAST1 protein homolog 1 OS=Arabidopsis thaliana OX=3702 GN=GASA1 PE=1 SV=1	11	1	1
F4I8B9	Putative WEB family protein At1g65010, chloroplastic OS=Arabidopsis thaliana OX=3702 GN=At1g65010 PE=1 SV=1	1	1	1
F4IGL7	Fructose-bisphosphate aldolase OS=Arabidopsis thaliana OX=3702 GN=FBA1 PE=1 SV=1	4	1	1

F4IJ45	Calcium-binding EF hand family protein OS=Arabidopsis thaliana OX=3702 GN=TCH3 PE=1 SV=1	6	1	1
F4IN36	Serine/threonine-protein phosphatase OS=Arabidopsis thaliana OX=3702 GN=PP2A-3 PE=1 SV=1	3	1	1
F4J6Z1	Ubiquitin E2 variant 1D-4 OS=Arabidopsis thaliana OX=3702 GN=UEV1D-4 PE=1 SV=1	9	1	1
O04482	Ubiquitin carboxyl-terminal hydrolase 2 OS=Arabidopsis thaliana OX=3702 GN=UCH2 PE=1 SV=1	2	1	1
O04487	Probable elongation factor 1-gamma 1 OS=Arabidopsis thaliana OX=3702 GN=At1g09640 PE=1 SV=1	5	2	2
O04603	50S ribosomal protein L5, chloroplastic OS=Arabidopsis thaliana OX=3702 GN=RPL5 PE=2 SV=1	3	1	1
O22683	Cyanate hydratase OS=Arabidopsis thaliana OX=3702 GN=CYN PE=2 SV=1	7	1	1
O24500	(S)-2-hydroxy-acid oxidase (Fragment) OS=Arabidopsis thaliana OX=3702 PE=2 SV=1	4	1	1
O49203	Nucleoside diphosphate kinase III, chloroplastic/mitochondrial OS=Arabidopsis thaliana OX=3702 GN=NDPK3 PE=1 SV=1	16	4	4
O65282	20 kDa chaperonin, chloroplastic OS=Arabidopsis thaliana OX=3702 GN=CPN20 PE=1 SV=2	13	3	3
O80504	10 kDa chaperonin 2, chloroplastic OS=Arabidopsis thaliana OX=3702 GN=CPN10-2 PE=2 SV=1	22	2	4
P10796	Ribulose bisphosphate carboxylase small chain 1B, chloroplastic OS=Arabidopsis thaliana OX=3702 GN=RBCS-1B PE=1 SV=1	23	4	5
P17745	Elongation factor Tu, chloroplastic OS=Arabidopsis thaliana OX=3702 GN=TUFA PE=1 SV=1	10	5	5
P29380	Cytochrome c OS=Arabidopsis thaliana OX=3702 GN=CC-1 PE=3 SV=1	6	1	1
P38418	Lipoxygenase 2, chloroplastic OS=Arabidopsis thaliana OX=3702 GN=LOX2 PE=1 SV=1	3	3	3
P42745	Ubiquitin-conjugating enzyme E2 2 OS=Arabidopsis thaliana OX=3702 GN=UBC2 PE=2 SV=1	11	1	1
P42761	Glutathione S-transferase F10 OS=Arabidopsis thaliana OX=3702 GN=GSTF10 PE=1 SV=3	9	2	2
P42825	Chaperone protein dnaJ 2 OS=Arabidopsis thaliana OX=3702 GN=ATJ2 PE=1 SV=2	4	1	1
P59259	Histone H4 OS=Arabidopsis thaliana OX=3702 GN=At1g07660 PE=1 SV=2	21	2	3
P61841	30S ribosomal protein S7, chloroplastic OS=Arabidopsis thaliana OX=3702 GN=rps7-A PE=1 SV=1	6	1	1

Q0WRA6	Histone H2A OS=Arabidopsis thaliana OX=3702 GN=At4g27230 PE=2 SV=1	12	2	2
Q1H5F2	Histone H2B OS=Arabidopsis thaliana OX=3702 PE=2 SV=1	15	2	2
Q38845	Serine/threonine-protein phosphatase 2A 65 kDa regulatory subunit A alpha isoform OS=Arabidopsis thaliana OX=3702 GN=PP2AA1 PE=1 SV=1	3	1	1
Q39086	Receptor-like serine/threonine-protein kinase SD1-7 OS=Arabidopsis thaliana OX=3702 GN=SD17 PE=1 SV=1	1	1	1
Q3ZVB1	PSII reaction center subunit V (Fragment) OS=Arabidopsis thaliana OX=3702 GN=psbE PE=4 SV=1	16	1	1
Q42214	Ubiquitin (Fragment) OS=Arabidopsis thaliana OX=3702 PE=2 SV=1	28	2	4
Q42276	Chlorophyll a-b binding protein, chloroplastic (Fragment) OS=Arabidopsis thaliana OX=3702 PE=2 SV=1	20	2	2
Q42291	Glycine hydroxymethyl transferase (Fragment) OS=Arabidopsis thaliana OX=3702 PE=2 SV=1	14	1	1
Q42406	Peptidyl-prolyl cis-trans isomerase CYP18-4 OS=Arabidopsis thaliana OX=3702 GN=CYP18-4 PE=1 SV=1	5	1	1
Q42449	Profilin-1 OS=Arabidopsis thaliana OX=3702 GN=PRF1 PE=1 SV=1	7	1	1
Q42589	Non-specific lipid-transfer protein 1 OS=Arabidopsis thaliana OX=3702 GN=LTP1 PE=2 SV=1	19	2	2
Q540F4	Ferredoxin-dependent glutamate synthase OS=Arabidopsis thaliana OX=3702 PE=3 SV=1	1	1	1
Q56X28	Putative phosphoglucomutase OS=Arabidopsis thaliana OX=3702 GN=At1g23190 PE=2 SV=1	5	1	1
Q56XI5	Cysteine proteinase RD21A OS=Arabidopsis thaliana OX=3702 GN=At1g47128 PE=2 SV=1	3	2	2
Q56Z17	Carboxyltransferase alpha subunit OS=Arabidopsis thaliana OX=3702 GN=At2g38040 PE=2 SV=1	6	1	1
Q7M1X0	Glycine cleavage system protein H1 and H2 (Fragment) OS=Arabidopsis thaliana OX=3702 PE=1 SV=1	53	1	1
Q84XU2	Serine/threonine-protein phosphatase 5 OS=Arabidopsis thaliana OX=3702 GN=PAPP5 PE=1 SV=1	2	1	1
Q85B88	Ribulose bisphosphate carboxylase large chain (Fragment) OS=Arabidopsis thaliana OX=3702 GN=RBCL PE=3 SV=1	48	22	28
Q8H135	ATP synthase subunit beta (Fragment) OS=Arabidopsis thaliana OX=3702 GN=At5g08670 PE=2 SV=1	7	3	3
Q8HT11	Photosystem II CP43 reaction center protein (Fragment) OS=Arabidopsis thaliana OX=3702 GN=psbC PE=3	14	5	5

	SV=1			
Q8HT12	Photosystem II D2 protein (Fragment) OS=Arabidopsis thaliana OX=3702 GN=psbD PE=3 SV=1	6	2	3
Q8LAZ4	Nitrilase 3 OS=Arabidopsis thaliana OX=3702 PE=2 SV=1	5	2	2
Q8LBB8	Putative arginase OS=Arabidopsis thaliana OX=3702 PE=2 SV=1	10	3	3
Q93XW5	Nitrile-specifier protein 5 OS=Arabidopsis thaliana OX=3702 GN=NSP5 PE=2 SV=1	7	2	2
Q944A7	Serine/threonine-protein kinase BSK1 OS=Arabidopsis thaliana OX=3702 GN=BSK1 PE=1 SV=1	3	1	1
Q944M3	Phosphopyruvate hydratase OS=Arabidopsis thaliana OX=3702 GN=At2g36530 PE=2 SV=1	3	1	1
Q945A8	2-oxoglutarate-dependent dioxygenase (Fragment) OS=Arabidopsis thaliana OX=3702 GN=AOP2 PE=2 SV=1	1	1	1
Q948K6	Ras-related protein RABG1 OS=Arabidopsis thaliana OX=3702 GN=RABG1 PE=2 SV=1	5	1	1
Q94AM2	Putative dihydrolipoamide S-acetyltransferase (Fragment) OS=Arabidopsis thaliana OX=3702 GN=At3g25860 PE=2 SV=2	4	1	1
Q9FH46	Signal recognition particle subunit SRP68 OS=Arabidopsis thaliana OX=3702 GN=At5g61970 PE=1 SV=1	1	1	1
Q9FI56	Chaperone protein ClpC1, chloroplastic OS=Arabidopsis thaliana OX=3702 GN=CLPC1 PE=1 SV=1	6	5	5
Q9FLP6	Small ubiquitin-related modifier 2 OS=Arabidopsis thaliana OX=3702 GN=SUMO2 PE=1 SV=1	16	1	1
Q9FPT1	Ubiquitin C-terminal hydrolase 12 OS=Arabidopsis thaliana OX=3702 GN=UBP12 PE=1 SV=2	2	1	1
Q9LJE4	Chaperonin 60 subunit beta 2, chloroplastic OS=Arabidopsis thaliana OX=3702 GN=CPN60B2 PE=1 SV=1	16	10	13
Q9LS03	Allene oxide cyclase 1, chloroplastic OS=Arabidopsis thaliana OX=3702 GN=AOC1 PE=1 SV=1	4	1	1
Q9LTX9	Heat shock 70 kDa protein 7, chloroplastic OS=Arabidopsis thaliana OX=3702 GN=HSP70-7 PE=2 SV=1	14	11	12
Q9LU86	Peroxiredoxin Q, chloroplastic OS=Arabidopsis thaliana OX=3702 GN=PRXQ PE=1 SV=1	3	1	1
Q9M1X3	AT3g63160/F16M2_10 OS=Arabidopsis thaliana OX=3702 GN=F16M2_10 PE=1 SV=1	13	1	1
Q9S7M0	Chlorophyll a-b binding protein 3, chloroplastic OS=Arabidopsis thaliana OX=3702 GN=LHCB3 PE=1 SV=1	3	1	1
Q9SL15	Glycine-rich protein 3 OS=Arabidopsis thaliana OX=3702 GN=GRP3 PE=1 SV=1	8	1	1

Q9SN86	Malate dehydrogenase, chloroplastic OS=Arabidopsis thaliana OX=3702 GN=At3g47520 PE=1 SV=1	3	1	1
Q9SYL9	50S ribosomal protein L13, chloroplastic OS=Arabidopsis thaliana OX=3702 GN=RPL13 PE=2 SV=1	4	1	1
Q9T043	60S ribosomal protein L14-2 OS=Arabidopsis thaliana OX=3702 GN=RPL14B PE=1 SV=1	7	1	1
Q9ZUC1	NADPH-dependent alkenal/one oxidoreductase, chloroplastic OS=Arabidopsis thaliana OX=3702 GN=AOR PE=1 SV=2	3	1	1
