# Supplementary material for: Hydroxylation of HPPD facilitates its PUB11-mediated ubiquitination and degradation in response to oxidative stress in Arabidopsis
Source: Plant Commun. 2025 Sep 8;6(11):101521. doi: 10.1016/j.xplc.2025.101521 (PMC12785156; doi:10.1016/j.xplc.2025.101521)
Supplement: Document S2. Article plus supplemental information [file mmc3.pdf]

Plant Communications
Research article

CellPress
Partner Journal

Hydroxylation of HPPD facilitates its PUB11-mediated ubiquitination and degradation in response to oxidative stress in *Arabidopsis*

Xin-He Yu[1,2,7], Xun Wen[1,2,7], Jiangqing Dong[3,4,7], Ya-Fang Hu[1,2], Xin-Long Wang[1,2], Dan-Yi Zhu[1,2], Qihua Ling[5,6,*], Hong-Yan Lin[1,2,*] and Guang-Fu Yang[1,2,*]

[1]State Key Laboratory of Green Pesticide, Central China Normal University, Wuhan 430079, P.R. China

[2]International Joint Research Center for Intelligent Biosensor Technology and Health, Central China Normal University, Wuhan 430079, P.R. China

[3]Hubei Shizhen Laboratory, Wuhan 430061, P.R. China

[4]School of Basic Medical Sciences, Hubei University of Chinese Medicine, Wuhan 430065, P.R. China

[5]Key Laboratory of Plant Carbon Capture, CAS Centre for Excellence in Molecular Plant Sciences, Institute of Plant Physiology and Ecology, Chinese Academy of Sciences, Shanghai 200032, China

[6]CAS-JIC Center of Excellence for Plant and Microbial Sciences (CEPAMS), Institute of Plant Physiology and Ecology, Chinese Academy of Sciences, Shanghai 200032, China

[7]These authors contributed equally to this article.

***Correspondence: Qihua Ling (qhling@cemps.ac.cn), Hong-Yan Lin (hylin@ccnu.edu.cn), Guang-Fu Yang (gfyang@ccnu.edu.cn)**

https://doi.org/10.1016/j.xplc.2025.101521

ABSTRACT

4-Hydroxyphenylpyruvate dioxygenase (HPPD) is critical for plant photosynthesis and essential for enhancing tolerance to oxidative stress. However, the precise mechanisms by which plants regulate HPPD in response to oxidative stress remain largely unknown. Here, we show that *Arabidopsis thaliana* HPPD (*At*HPPD) undergoes a previously uncharacterized post-translational modification—phenylalanine hydroxylation—in response to excessive hydroxyl radicals (·OH), thereby mediating oxidative stress tolerance. Biochemical analyses revealed that this hydroxylation impairs the normal function of *At*HPPD, accelerating its degradation. We further identified PUB11 as a key interactor of *At*HPPD. Both *in vitro* and *in vivo* assays demonstrated that this interaction is enhanced under oxidative stress, promoting ubiquitination and facilitating rapid *At*HPPD degradation via the 26S proteasome to maintain reactive oxygen species homeostasis. Overall, this work uncovers a novel mechanism by which plants balance photosynthetic efficiency with the repair of oxidative damage, identifies key processes in oxidative stress regulation, and provides a foundation for breeding crops with improved resilience to abiotic stress.

Key words: 4-hydroxyphenylpyruvate dioxygenase, HPPD, hydroxylation, oxidative stress, ubiquitination

Yu X.-H., Wen X., Dong J., Hu Y.-F., Wang X.-L., Zhu D.-Y., Ling Q., Lin H.-Y., and Yang G.-F. (2025). Hydroxylation of HPPD facilitates its PUB11-mediated ubiquitination and degradation in response to oxidative stress in *Arabidopsis*. Plant Comm. **6**, 101521.

INTRODUCTION

Abiotic stress, encompassing low nutrient availability, drought, salinity, extreme temperatures, toxic metals, and elevated UV radiation, consistently hampers plant growth and development and imposes significant constraints on global agricultural productivity (Zhang et al., 2022, Zhang et al., 2023a). Understanding how plants perceive and adapt to environmental stress is critical for ensuring global food security. Oxidative damage is a major consequence of abiotic stress in plants, disrupting numerous biological processes due to the excessive accumulation of reactive oxygen species (ROS) (Mangano et al., 2016; Xie et al., 2019). Plants respond to oxidative stress at multiple levels, including sensing, signaling, transcription, RNA processing, translation, and post-translational modifications (PTMs) (Zhu, 2016; Guccione et al., 2019; Li et al., 2021).

4-Hydroxyphenylpyruvate dioxygenase (HPPD), a vital enzyme in the non-heme Fe(II)/2-oxoacid-dependent oxygenase superfamily, is critical for the synthesis of essential plant metabolites such as vitamin E and plastoquinone (Islam et al., 2018; Lin et al., 2023; Yu et al., 2023). Suppression of HPPD-mediated carotenoid biosynthesis results in bleaching, necrosis, and ultimately cell death. Consequently, HPPD has been recognized as an attractive

 © 2025 The Author(s). Published by Elsevier Inc. on behalf of CAS Center for Excellence in Molecular Plant Sciences, Chinese Academy of Sciences, and Chinese Society for Plant Biology. This is an open access article under the CC BY license (http://creativecommons.org/licenses/by/4.0/).

herbicide target, spurring the development of numerous inhibitors (Wang et al., 2015; Lin et al., 2019; Yan et al., 2022). Recent studies have demonstrated that HPPD is involved in responses to abiotic stress, including salt, drought, and oxidative stress (Fu et al., 2022; Zeng et al., 2023). Stress conditions induce HPPD expression, which improves tolerance to abiotic challenges. For instance, heavy metal stress induces HPPD expression and elevates vitamin E levels in *Arabidopsis*, thereby enhancing oxidative stress tolerance (Tsegaye et al., 2002). Transgenic plants overexpressing HPPD also show enhanced tolerance to abiotic stress (Jiang et al., 2017; Kim et al., 2021). Overall, HPPD has multiple essential physiological functions in plants, and maintaining its stability is crucial for growth, development, and adaptation to environmental changes. However, the mechanisms by which plants regulate HPPD in response to abiotic stress remain largely unknown.

PTMs contribute significantly to plant stress responses by modulating protein stability and coordinating multiple signaling pathways (Han et al., 2023; Lee et al., 2023; Zhang et al., 2023a, 2023b). Extensive studies have shown that oxidative modifications alter protein function, thereby affecting signaling pathways during oxidative stress (Mekhail et al., 2004; Ruiz-May et al., 2019; Lee et al., 2023). Furthermore, homeostasis during oxidative stress is maintained by regulating the stability of modified proteins. Although PTMs of various proteins have been extensively studied in the context of abiotic stress, the specific PTMs of HPPD and their functional implications remain largely unexplored. Given HPPD's central role in the biosynthesis of plastoquinone, a component of the photosynthetic electron transport chain, it is crucial to investigate how HPPD is regulated under oxidative stress (Li et al., 2022). As chloroplasts are the primary site of ROS generation in plants, understanding HPPD's response to ROS signals such as H_2O_2 is essential. However, it remains unclear whether HPPD undergoes PTMs in response to oxidative stress. Identifying HPPD PTMs associated with abiotic stress is critical for understanding HPPD's role in stress resistance mechanisms.

In this study, we show that the phenylalanine hydroxylation level of *At*HPPD increases under oxidative stress, markedly reducing its enzymatic activity. Through an immunoprecipitation–mass spectrometry (IP–MS) screening assay, we identified the U-box E3 ligase PUB11 as an interactor of *At*HPPD. Biochemical analyses *in vitro* and *in vivo* further revealed that PUB11 facilitates the ubiquitination of *At*HPPD, leading to the rapid degradation of hydroxylated *At*HPPD via the 26S proteasome pathway. Notably, oxidative stress enhances the interaction between *At*HPPD and PUB11 through hydroxylation, thereby accelerating its turnover. This post-translational regulatory mechanism contributes to the maintenance of ROS homeostasis. Collectively, our findings uncover a previously uncharacterized PTM of HPPD that occurs in response to oxidative stress and highlight the pivotal role of the ubiquitin–proteasome system in plant stress tolerance.

RESULTS

Phenylalanine hydroxylation of HPPD in response to oxidative stress in *Arabidopsis*

To determine whether HPPD undergoes PTMs in response to oxidative stress, we treated *Arabidopsis* with 50 mM H_2O_2 for 24 h and immunoprecipitated lysates using a specific antibody against *At*HPPD (Supplemental Figure 1). The untreated sample served as a control. The immunoprecipitates were analyzed by liquid chromatography–tandem MS (LC–MS/MS) to identify modified peptides. We identified a peptide with the sequence SFF^{132}SSHGLGVR, which contains residue F132, and it exhibited a +15.995 Da shift at the b^3 ion in the *b*-ion series and the y^9 ion in the *y*-ion series (Figure 1A). Hydroxylation was also detected at F47, F52, F215, F419, and F428 (Supplemental Figures 2A–2E), but not at other phenylalanine residues such as F381. Interestingly, similar modifications were also observed in the control group, which is likely due to HPPD's known propensity for self-hydroxylation (Figure 1B) (Bradley et al., 1986; Liu et al., 2001). However, under oxidative stress, the abundance of modifications increased significantly at most sites, including F131, F132, F215, F419, and F428. In particular, hydroxylation of F428 increased from 0.80% to 6.0%, representing a 7.5-fold increase (Figure 1B). These observations, especially the significant increase in hydroxylation abundance at specific sites under oxidative stress, strongly suggest that oxidative stress is a key trigger for HPPD hydroxylation.

To further examine the relationship between HPPD hydroxylation and oxidative stress, we expressed *At*HPPD in *E. coli* BL21(DE3) and identified hydroxylated phenylalanine residues using trypsin digestion followed by nanoscale capillary LC–MS/MS. Analysis of the secondary chromatogram revealed a greater degree of modification than that observed *in vivo*. In addition to the previously identified sites, hydroxylation was also detected at F64 and F424 (Supplemental Figures 3A and 3B). Crystal structures of *At*HPPD (PDB: 7X5R, 7CQS, 7X5U, etc.) purified from *E. coli* also showed hydroxylation at F132 (Supplemental Figure 3C). The highest level of hydroxylation was 8.3% at F64; the levels at other residues included 0.7% at F424 and F428 (Figure 1C). Furthermore, the hydroxylation levels measured by LC–MS/MS at each site were similar *in vitro* and *in vivo* (Supplemental Figure 3D). To confirm that phenylalanine hydroxylation is induced by hydroxyl radicals, we assessed hydroxylation levels under 50 and 250 mM H_2O_2. A positive correlation was observed between oxidative stress intensity and modification levels at most phenylalanine residues (Figure 1C). Notably, F29 and F202 were hydroxylated in response to H_2O_2 (Figure 1C). The most significant changes were observed at F131 and F132, where the hydroxylation level increased from 0.14% without H_2O_2 to 1.64% with 50 mM H_2O_2, an 11.7-fold increase. At 250 mM H_2O_2, the modification abundance reached 4.53%, only a 2.8-fold further increase compared with 50 mM (Figure 1C). In contrast, the hydroxylation level of F419 showed no significant variation, which suggests that hydroxylation levels across different sites may differ, possibly influenced by the local environment of the phenylalanine residue (Figure 1C).

To further assess the specificity of hydroxylation at these residues, we performed site-directed mutagenesis of HPPD at the identified residues (F29, F47, F52, F64, F131, F132, F202, F215, F419, F424, and F428) (Supplemental Figure 3E) and carried out hydroxylation assays on the mutants. LC–MS/MS analysis showed that when these phenylalanine residues were mutated to alanine (A), no hydroxylation was detected (Supplemental Table 1). Overall, these results demonstrate that phenylalanine residues in HPPD undergo hydroxylation under

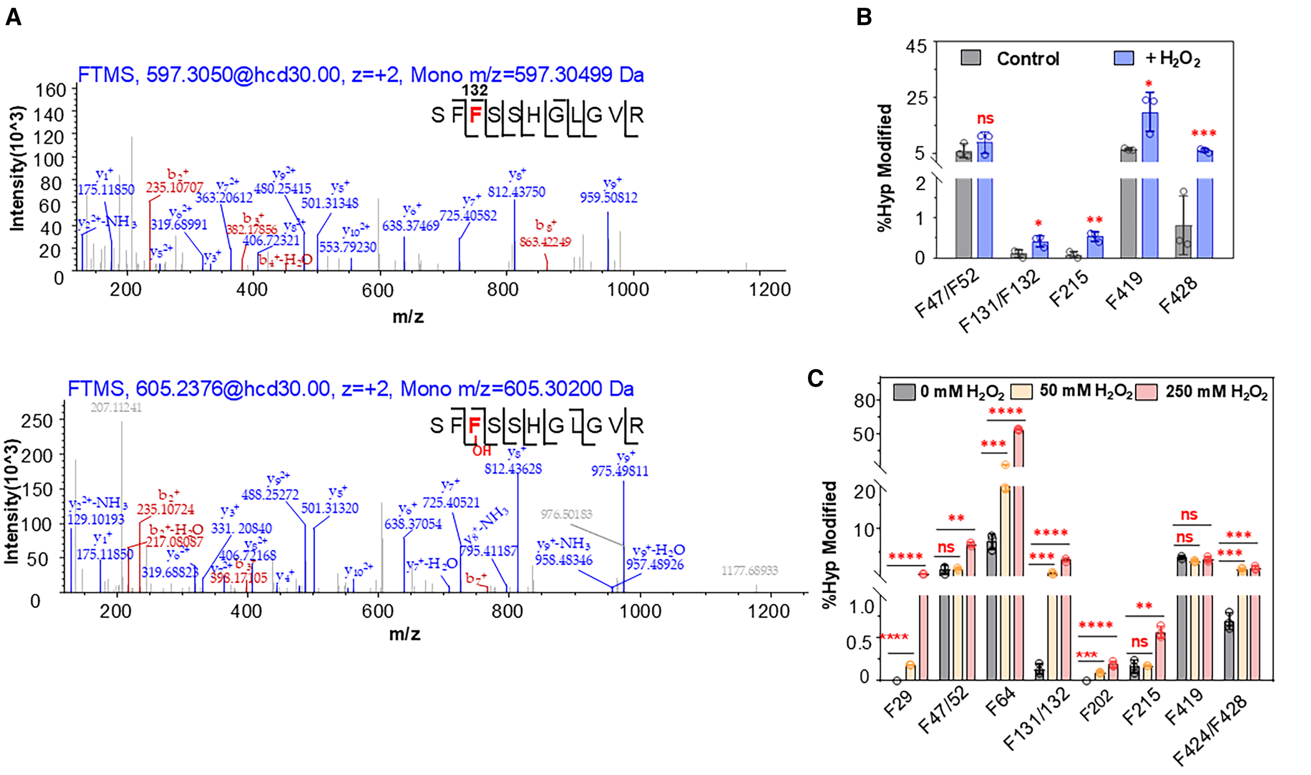

Figure 1. Relationship between HPPD hydroxylation and oxidative stress.

(A) *At*HPPD is hydroxylated at F132. The product ion spectrum of the endogenous doubly charged ion at *m/z* 605.2375 Th corresponds to the hydroxylated peptide SFF(OH)SSHGLGVR, showing *b*- and *y*-fragment ions typical of higher-energy C-trap dissociation fragmentation, enabling peptide sequence identification.

(B) Correlation between hydroxylation levels and oxidative stress *in vivo*.

(C) Correlation between hydroxylation levels and oxidative stress *in vitro*.

$^{*}p < 0.05$, $^{**}p < 0.01$, $^{***}p < 0.001$, $^{****}p < 0.0001$, and ns: not significant. %Hyp refers to the level of hydroxylation. Error bars indicate ± standard deviation (SD).

oxidative stress and that this hydroxylation response is highly residue specific.

Phenylalanine hydroxylation-induced HPPD degradation

Hydroxylation generally modulates protein stability (Ivan et al., 2001; Mekhail et al., 2004). To determine whether hydroxylation affects the stability of HPPD, we conducted an *in vitro* cell-free degradation assay. An immunoblot assay using an anti-His antibody was performed to measure the abundance of His-tagged *At*HPPD. The *At*HPPD protein was unstable in wild-type (WT) *Arabidopsis thaliana* protein extract and was clearly degraded after 1 h, becoming almost completely depleted by 2 h without H_2O_2 treatment (Figure 2A). *At*HPPD exhibited pronounced degradation beginning at 30 min when treated with 250 mM H_2O_2, resulting in substantial degradation within 1 h under oxidative stress conditions (Figures 2A and 2B). Nevertheless, H_2O_2 alone could not directly degrade *At*HPPD (Supplemental Figure 4). Furthermore, the degradation rate of *At*HPPD in 250 mM H_2O_2 was significantly accelerated compared with that observed in 50 mM H_2O_2 (Figures 2A and 2B; Supplemental Figure 5). To assess the turnover rate of *At*HPPD *in vivo*, we treated seedlings with cycloheximide (CHX; 100 μM), an inhibitor of protein biosynthesis. Because *At*HPPD levels are low under normal growth conditions, we used GFP-tagged, *At*HPPD-overexpressing (*At*HPPD-GFP-OE) transgenic *Arabidopsis* seedlings (Supplemental Figure 6). An anti-GFP antibody immunoblot was used to measure *At*HPPD-GFP protein levels. As shown in Figures 2C–2F, *At*HPPD remained relatively stable in seedlings when treated with CHX alone. In contrast, combined treatment with CHX and H_2O_2 induced rapid *At*HPPD degradation, which was consistent with the results from the cell-free degradation assay (Figures 2A–2D and 2F). This degradation is attributable to oxidative stress-induced hydroxylation of *At*HPPD, as confirmed in Figure 1C.

To investigate the relationship between hydroxylation and degradation, we treated hydroxylated *At*HPPD with the ROS scavenger N-acetylcysteine (NAC) in a cell-free degradation assay. NAC treatment markedly suppressed *At*HPPD degradation within 1.5 h (Figures 2G and 2H). We then examined the hydroxylation levels of *At*HPPD with and without NAC treatment and found that they were significantly reduced at all sites except F215, F424, and F428 (Figure 2I). Overall, these results suggest that hydroxylation is the primary driver of *At*HPPD degradation.

Because hydroxylation is known to alter protein activity, we investigated its effects on *At*HPPD function by kinetically characterizing HPPD under oxidative stress. The Michaelis–Menten constants (K_m) and catalytic constants (k_{cat}) for WT and

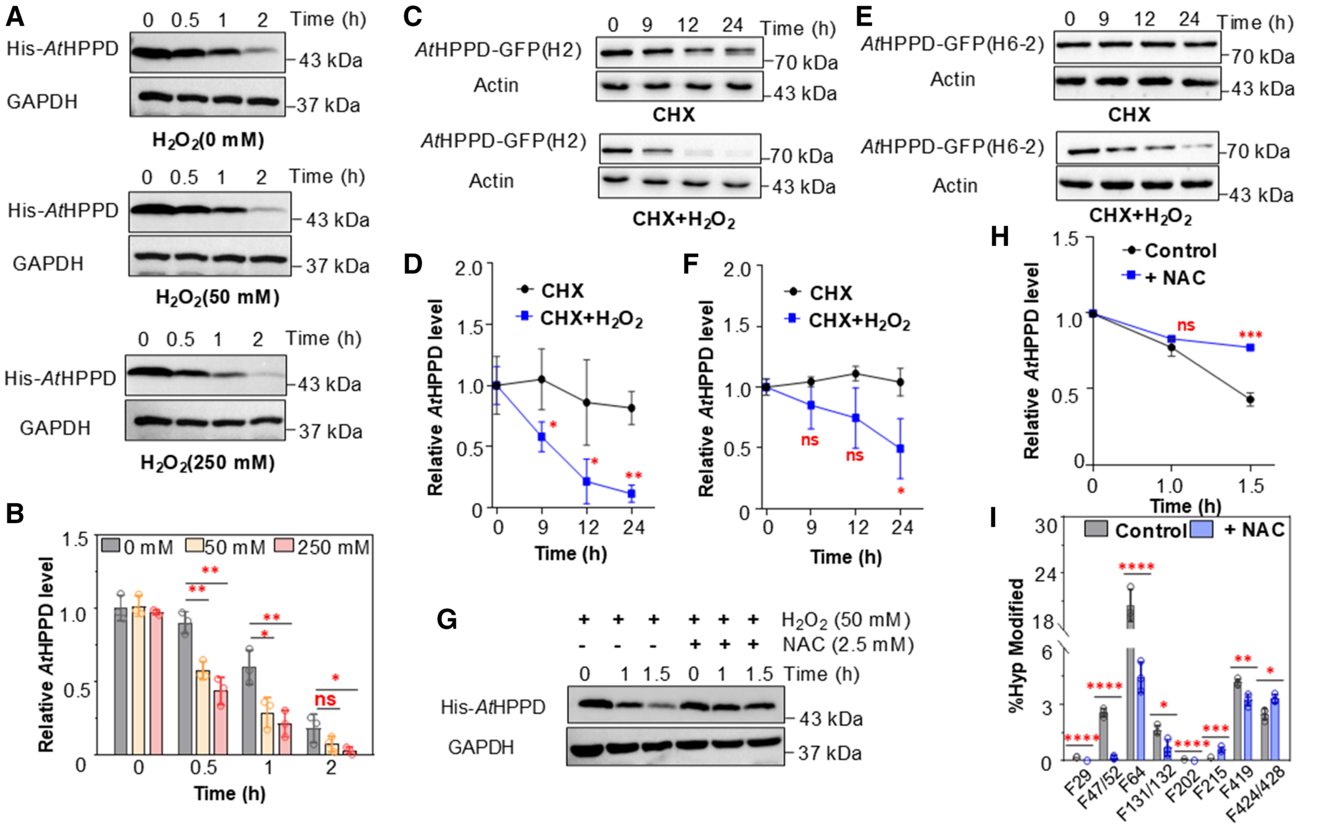

Figure 2. Hydroxylation of phenylalanine promotes *At*HPPD degradation.

(A) *In vitro* cell-free degradation assay showing *At*HPPD degradation in the presence of 0, 50, and 250 mM H_2O_2.

(B) Relative His-*At*HPPD band intensity from **(A)**, normalized to 0 h using Touch View. Data are means of three replicates, and individual results for each replicate are shown. Significant differences compared with 0 h were determined using Student's *t*-test: $^*p < 0.05$, $^{**}p < 0.01$, and ns: not significant.

(C) Effect of H_2O_2 on *At*HPPD stability. Cell lysates from 10-day-old seedlings overexpressing *At*HPPD-GFP (line H2) were treated with 100 μM translation inhibitor CHX and H_2O_2. Reactions were stopped at the indicated time points and analyzed by immunoblotting with an anti-GFP antibody.

(D) Relative GFP-*AtHPPD* band intensity from **(C)**, normalized to 0 h using Touch View. Data are means of three replicates, and individual results for each replicate are shown.

(E) Effect of H_2O_2 on *At*HPPD stability. Cell lysates from 10-day-old seedlings overexpressing *At*HPPD-GFP (line H6-2) were treated with 100 μM translation inhibitor CHX and H_2O_2. Reactions were stopped at the indicated time points and analyzed by immunoblotting with an anti-GFP antibody.

(F) Relative GFP-*At*HPPD band intensity from **(E)**, normalized to 0 h using Touch View. Data are means of three replicates, and individual results for each replicate are shown.

(G) Effect of NAC on *At*HPPD stability in an *in vitro* cell-free degradation assay.

(H) Relative His-*At*HPPD band intensity from **(G)**, normalized to 0 h using Touch View. Data are means of three replicates, and individual results for each replicate are shown.

(I) Hydroxylation levels of *At*HPPD after NAC treatment. $^{***}p < 0.001$ and $^{****}p < 0.0001$. Error bars indicate ± standard deviation (SD).

hydroxylated *At*HPPD were measured (Supplemental Table 2). Notably, upon exposure to 250 mM H_2O_2, the binding affinity of *At*HPPD for HPPA decreased by approximately six-fold compared to the WT. In addition, exposure to 50 and 250 mM H_2O_2 resulted in 2.90- and 2.63-fold decreases in k_{cat}, respectively. The k_{cat}/K_m values showed an overall decline compared with the WT; in particular, a 16.17-fold decrease was observed under 250 mM H_2O_2.

The results described above suggest that HPPD contains 11 phenylalanine residues with the potential for hydroxylation. To identify which residues most significantly affect its activity, we performed site-directed mutagenesis on *At*HPPD and kinetically characterized the mutants. Hydroxylation converts phenylalanine to tyrosine (Zhang et al., 2011); therefore, we introduced point mutations to tyrosine at the hydroxylation sites and overexpressed these mutants in *E. coli* BL21(DE3) to evaluate their activity. As shown in Table 1, all mutants exhibited sharp decreases in k_{cat}/K_m values compared with the WT, which indicates that hydroxylation disrupts *At*HPPD's normal biological functions. The mutants F419Y and F424Y showed the greatest decrease, losing catalytic activity entirely, demonstrating that they are essential for normal function. Circular dichroism spectroscopy of the WT and its mutants showed no significant alterations in secondary structure, ruling out structural perturbation as the basis for their reduced activity (Supplemental Figure 7).

Overall, these results suggest that H_2O_2 induces hydroxylation of *At*HPPD, which significantly reduces *At*HPPD enzymatic activity and disrupts its normal biological function. The impaired HPPD is then rapidly degraded, which may help maintain normal plant growth.

Enzyme	K_m (μM)	k_{cat} (s^{-1})	k_{cat}/K_m (s^{-1} μM^{-1})
*At*HPPD-F29Y	1.340 ± 0.076	0.351 ± 0.008	0.262
*At*HPPD-F47Y	1.098 ± 0.040	0.351 ± 0.008	0.199
*At*HPPD-F52Y	1.304 ± 0.053	0.074 ± 0.002	0.057
*At*HPPD-F64Y	1.268 ± 0.049	0.136 ± 0.001	0.107
*At*HPPD-F131Y	2.053 ± 0.097	0.277 ± 0.017	0.135
*At*HPPD-F132Y	2.116 ± 0.064	0.782 ± 0.015	0.369
*At*HPPD-F202Y	1.465 ± 0.059	0.450 ± 0.030	0.307
*At*HPPD-F215Y	2.063 ± 0.198	0.094 ± 0.012	0.045
*At*HPPD-F419Y	N/A	N/A	N/A
*At*HPPD-F424Y	N/A	N/A	N/A
*At*HPPD-F428Y	7.977 ± 0.388	0.619 ± 0.002	0.078
*At*HPPD-WT	1.254 ± 0.090	0.864 ± 0.028	0.689

Table 1. Comparison of apparent catalytic activities for *AtHPPD* WT and mutants.
Each experiment was carried out in triplicate. N/A, not available.

Identification of the U-box E3 ligase PUB11 as an *AtHPPD* interaction partner

Previous research has demonstrated that hydroxylated proteins interact with E3 ligases, facilitating their degradation (Mekhail et al., 2004; Fong et al., 2008; Li et al., 2023). To identify E3 ligases that interact with *At*HPPD, we used Co-IP combined with LC–MS/MS (IP–MS) analysis. Total proteins from seedlings overexpressing *At*HPPD-GFP were extracted and subjected to IP using anti-GFP agarose. Proteins isolated from *Arabidopsis* seedlings overexpressing GFP alone served as the control. The samples were subsequently analyzed by LC–MS/MS. Proteins were qualitatively analyzed using data-dependent acquisition proteomics. A total of 194 high-confidence proteins were identified after filtering against the control (Supplemental Table 3).

As illustrated in Supplemental Figure 8, Gene Ontology (GO) classification and enrichment analysis of the identified proteins were performed for cellular components, molecular functions, and biological processes using resource for ontology analysis and discovery (ROAD) searching. The proteins were classified into 10 significantly enriched GO categories (hypergeometric $p < 0.05$) (Supplemental Figure 8A), including "cell part," "intracellular part," "intracellular," and "intracellular organelle." Based on GO annotation, the proteins were also classified into 10 molecular function categories (hypergeometric $p < 0.05$) (Supplemental Figure 8A), particularly organic cyclic compound binding (the most prevalent), heterocyclic compound binding, protein binding, and ion binding. Classification by biological processes (hypergeometric $p < 0.05$) showed the most frequent annotation as "cellular metabolic process" (Supplemental Figure 8A). In total, the 194 identified proteins were enriched in categories such as protein binding, photorespiration, glycolysis, and response to stress (Supplemental Figure 8B). Among these, the E3 ligase PUB11 was identified as a unique interaction partner of *At*HPPD (Supplemental Table 3). Because PUB11 is a well-established stress-related protein (Chen et al., 2021), we hypothesized that it may contribute to *At*HPPD degradation during abiotic stress responses.

To test this hypothesis, we first assessed the physical interaction between *At*HPPD and PUB11 using a yeast two-hybrid (Y2H) assay. The interaction observed in the Y2H system is shown in Figure 3A. *At*HPPD was fused to the DNA-binding domain, and full-length PUB11 was fused to the activation domain. Positive clones were observed on TDO/X selection medium, indicating that a specific interaction occurred between *At*HPPD and PUB11 in yeast cells. In contrast, negative controls showed no growth under identical conditions.

To identify the PUB11 region responsible for interaction with *At*HPPD, we divided PUB11 into three truncated variants and purified them from *Escherichia coli* extracts to assess their capacity to bind His-*At*HPPD through *in vitro* pull-down assays (Figure 3B). His-*At*HPPD was clearly pulled down by full-length MBP-PUB11 (detected with an anti-His antibody) but not by MBP alone (Figure 3C). Analysis of *At*HPPD and PUB11 deletion derivatives revealed that both the PUB11 U-box and armadillo repeat (ARM) repeats interact with *At*HPPD, with the ARM repeats exhibiting a stronger interaction (Figure 3C). Subsequently, we examined the direct interaction between PUB11 and *At*HPPD using a microscale thermophoresis (MST) assay (Figure 3D). Glutathione S-transferase (GST) proteins at identical concentrations served as negative controls. The binding affinity (K_d) of the interaction between GST-PUB11-ARM and His-*At*HPPD was 7.42 ± 1.42 μM. Notably, the interaction was stronger under oxidative stress, with a K_d of 2.93 ± 0.50 μM. We then measured the binding affinity of each of the 11 hydroxylation-site mutants with the GST-PUB11-ARM domain and found that most exhibited significantly stronger interactions than the WT protein (Supplemental Figure 9). These results suggest that hydroxylation may promote the degradation of HPPD by enhancing its interaction with PUB11.

The physical interaction between PUB11 and *At*HPPD was further confirmed using bimolecular fluorescence complementation (BiFC) in *N. benthamiana* leaves. PUB11 and *At*HPPD were fused to the C-terminal or N-terminal fragments of yellow fluorescent protein (YFP), generating PUB11-cYFP and *At*HPPD-nYFP, respectively. Co-infiltration of these two fusion

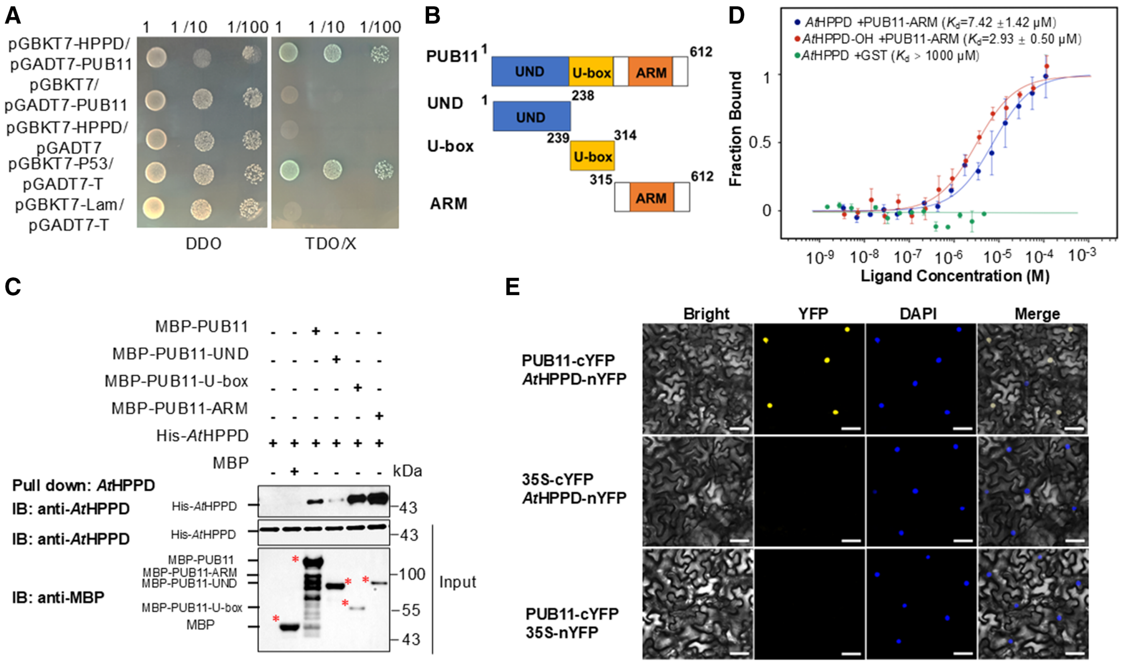

Figure 3. Identification of PUB11 as an interaction partner of *At*HPPD.

(A) *At*HPPD interacts with PUB11 in yeast. Yeast cells were grown on −DDO (−Leu−Trp) and −TDO/X (−His/−Leu/−Trp/X-gal) medium. pGBKT7-P53/pGADT7-T was used as the positive control, and pGBKT7-Lam/pGADT7-T as the negative control.

(B) Schematic diagrams of full-length PUB11 and the deletion variants used in the *in vitro* pull-down assays.

(C) Pull-down assays showing that PUB11 interacts with *At*HPPD. IB, immunoblotting.

(D) MST assay showing the interaction between *At*HPPD and PUB11. His-NTA dye-labeled *At*HPPD was incubated with varying concentrations of GST-PUB11-ARM for 20 min to assess binding affinity. Experiments were repeated three times, and error bars indicate ± standard deviation (SD).

(E) BiFC assays in *N. benthamiana* leaves showing the interaction between *At*HPPD-nYFP (nYFP) and PUB11-cYFP (cYFP). DAPI-stained DNA serves as a nuclear marker. Scale bars: 25 μm.

proteins in leaf epidermal cells led to strong YFP fluorescence, whereas control experiments co-expressing either PUB11-cYFP or *At*HPPD-nYFP with empty vectors showed no detectable YFP fluorescence (Figure 3E). Importantly, PUB11 interacted with *At*HPPD in the nucleus, as indicated by the co-localization of the BiFC signal with DAPI-stained nuclear markers. To gain a more comprehensive understanding of the co-localization patterns of PUB11 and *At*HPPD, we first examined their respective subcellular localizations using *Arabidopsis* protoplasts expressing GFP-fusion proteins (Supplemental Figure 10). *At*HPPD-GFP exhibited strong fluorescence predominantly in the cytoplasm, whereas PUB11-GFP displayed a strong nuclear signal accompanied by weaker signals in the cytoplasm. Subsequently, co-localization was tested by co-expressing PUB11-GFP and *At*HPPD-mCherry in *Arabidopsis* protoplasts. Co-localization was predominantly observed within the nucleus, which suggests that PUB11 may facilitate the nuclear translocation of HPPD for degradation (Supplemental Figure 11). Overall, these results provide strong evidence for the physical interaction between *At*HPPD and PUB11 in the nucleus.

PUB11-mediated degradation of *At*HPPD through the 26S proteasome to maintain ROS balance

Proteolysis is essential for the quality control of key regulatory proteins in plants (Smalle et al., 2004). Our protein interaction assays revealed that the E3 ligase PUB11 physically interacts with *At*HPPD, leading us to hypothesize a role in the ubiquitination and subsequent degradation of *At*HPPD. To investigate this, we first assessed the regulation of *At*HPPD by the 26S proteasome. Immunoblotting of *At*HPPD-GFP-OE seedlings treated with ATP (given that ubiquitination requires ATP hydrolysis to provide energy for peptide bond formation) showed that *At*HPPD was stable without ATP, whereas ATP resulted in rapid degradation (Figures 4A and 4B; Supplemental Figure 12). As further confirmation, an *in vitro* cell-free degradation assay was performed using WT *A. thaliana* plants. We used immunoblotting to measure *At*HPPD levels following treatment with MG132 (a 26S proteasome inhibitor); treatment with 50 μM MG132 significantly inhibited *At*HPPD degradation. These results suggest that the ubiquitin–proteasome system is involved in *At*HPPD degradation (Figures 4B and 4C).

To determine whether PUB11 mediates *At*HPPD ubiquitination and degradation, we assessed *At*HPPD degradation with or without PUB11 in a cell-free degradation assay. The presence of PUB11 significantly increased the rate of *At*HPPD degradation compared with its absence (Figure 4D; Supplemental Figure 13). To further investigate the role of PUB11 in plants, we identified homozygous T2 *pub11 A. thaliana* plants using Sanger sequencing (Supplemental Figure 14). The degradation rate of *At*HPPD was significantly lower in extracts from *pub11* plants than in those from WT plants (Figures 4D–4F); however, the addition of PUB11 protein markedly accelerated its degradation (Figure 4E; Supplemental Figure 15). These results indicate that PUB11 promotes the degradation of *At*HPPD *in vitro*. To more

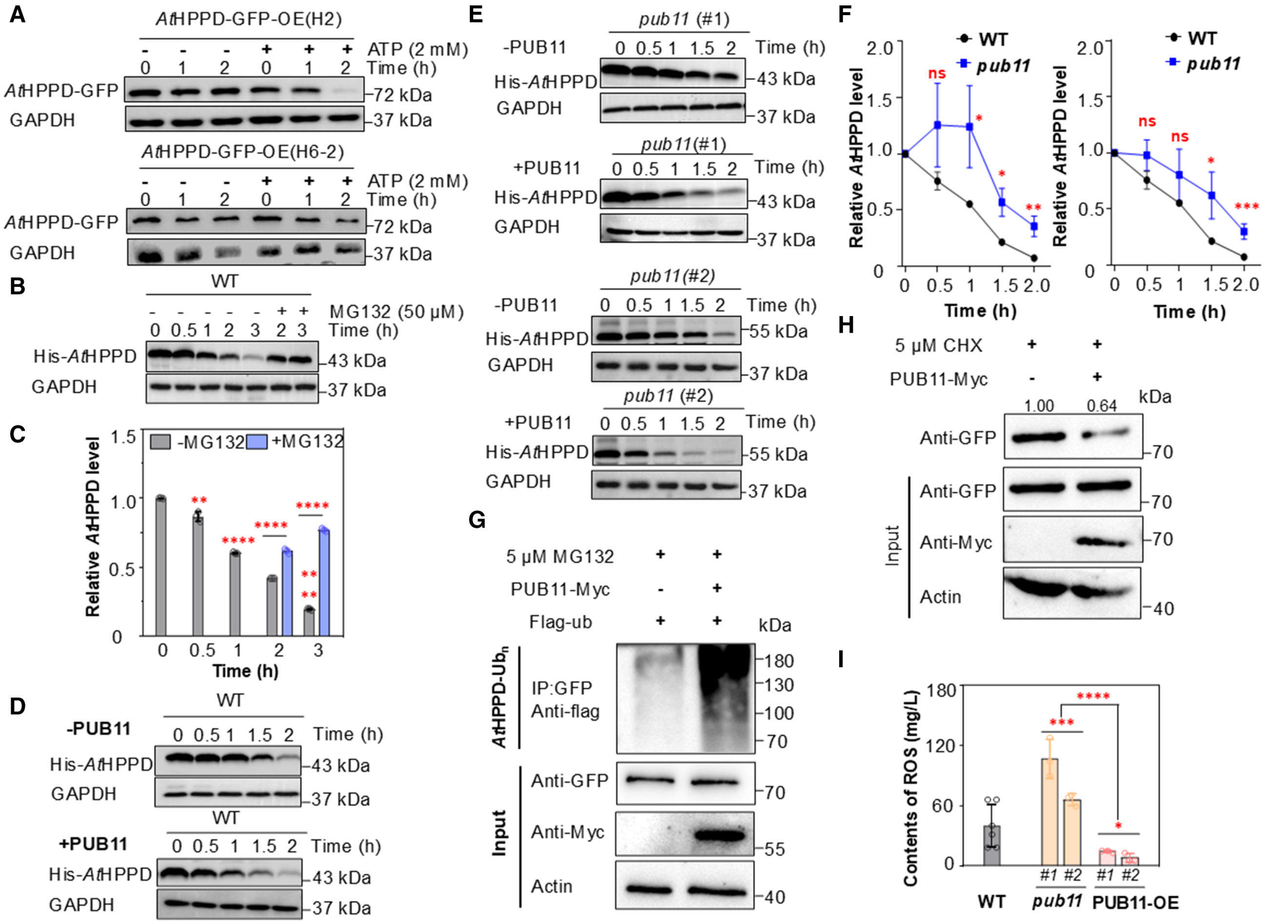

Figure 4. PUB11 promotes the ubiquitination and degradation of *At*HPPD to maintain ROS balance.

(A) *At*HPPD degradation is enhanced by ATP. Total proteins from *At*HPPD-GFP-OE seedlings (lines H2 and H6-2) were isolated and incubated with or without 2 mM ATP for various durations, followed by immunoblotting with an anti-GFP antibody. GAPDH served as a loading control.

(B) *At*HPPD degradation likely occurs primarily via the 26S proteasome. For MG132 treatment, WT *Arabidopsis thaliana* extracts were treated with 50 μM MG132 for 1 h and incubated with His-*At*HPPD for the specified durations. GAPDH served as a loading control.

(C) Relative His-*At*HPPD band intensity from **(B)**, normalized to 0 h using Touch View. Data are means of three replicates, and individual results for each replicate are shown. Significant differences compared with 0 h were determined using Student's *t*-test: $^{**}p < 0.01$ and $^{****}p < 0.0001$.

(D) *In vitro* cell-free degradation assays of His-*At*HPPD in protein extracts from WT plants with or without PUB11 protein.

(E) *In vitro* cell-free degradation assays of His-*At*HPPD in protein extracts from *pub11* plants with or without PUB11 protein.

(F) Time curves of His-*At*HPPD degradation rates for both WT and *pub11* **(D and E)**.

(G) PUB11 promotes the ubiquitination of *At*HPPD *in vivo*. Immunoprecipitated proteins were analyzed using an anti-FLAG antibody. *Arabidopsis* protoplasts were treated with 5 μM MG132 for 4 h prior to extraction.

(H) *In vivo* degradation experiments showing that PUB11 degrades *At*HPPD. *Arabidopsis* protoplasts were treated with 5 μM CHX for 6 h prior to extraction.

(I) Detection of leaf ROS levels via an enzyme-linked immunosorbent assay (ELISA) in the indicated *Arabidopsis thaliana* lines. Student's *t*-test: $^{*}p < 0.05$ and $^{***}p < 0.001$.

directly examine PUB11-mediated ubiquitination of *At*HPPD, we performed an *in vivo* ubiquitination assay (Ling et al., 2012). GFP-HPPD-OE *Arabidopsis* protoplasts were transiently transfected with the *Flag-Ub* gene, with or without the *Myc-PUB11* gene. We then immunoprecipitated GFP-HPPD from extracted proteins using an anti-GFP antibody and probed the eluted proteins with an anti-FLAG antibody. In *Arabidopsis* protoplasts overexpressing PUB11, ubiquitinated *At*HPPD levels were significantly higher than in those lacking PUB11 (Figure 4G). *In vivo* degradation assays further demonstrated that PUB11 promotes *At*HPPD degradation (Figure 4H). To confirm that PUB11 interacts with and promotes *At*HPPD degradation, we measured the levels of tocopherol, a downstream product of *At*HPPD. We generated PUB11-OE lines (Supplemental Figure 16) and quantified tocopherol levels in *pub11* mutants, PUB11-OE lines, and WT *A. thaliana* plants. Tocopherol levels were significantly reduced in PUB11-OE lines compared to both *pub11* mutants and WT (Supplemental Figure 17). Collectively, these results suggest that PUB11 promotes the ubiquitination and degradation of *At*HPPD both *in vitro* and *in vivo*, which may constitute a mechanism for *At*HPPD degradation under oxidative stress conditions.

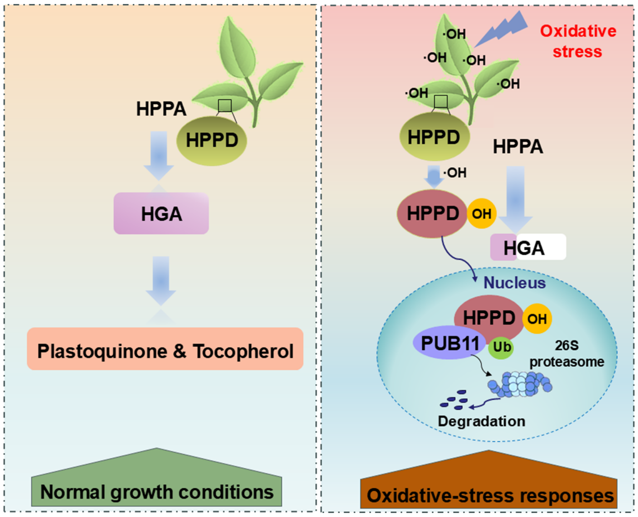

Figure 5. A proposed working model for HPPD-mediated oxidative stress response in *Arabidopsis*.

Under normal growth conditions, *At*HPPD catalyzes the conversion of HPPA to HGA, a crucial precursor for the biosynthesis of plastoquinone and tocopherol. However, under oxidative stress, excess hydroxyl radicals induce *At*HPPD hydroxylation. This modification impairs *At*HPPD's biological function and promotes its interaction with the E3 ligase PUB11. The interaction facilitates degradation of hydroxylated *At*HPPD in the nucleus via the 26S proteasome. HPPA, 4-hydroxyphenylpyruvate; HGA, homogentisic acid.

Given that PUB11 facilitates *At*HPPD degradation, we next investigated its role in the oxidative stress response. We quantified ROS levels in WT, *pub11* mutants, and PUB11-OE plants subjected to oxidative stress for 36 h using an enzyme-linked immunosorbent assay with absorbance measured at 450 nm. PUB11-OE plants exhibited a marked reduction in ROS accumulation relative to *pub11* mutants, with levels even lower than those in WT plants (Figure 4I). Overall, PUB11-mediated *At*HPPD degradation reduces intracellular ROS accumulation, thereby enhancing oxidative stress tolerance.

DISCUSSION

Photosynthesis is essential for plant growth and survival, but its limited efficiency in harnessing sunlight can generate harmful by-products, including ROS, which are both toxic and serve as crucial intracellular signaling molecules. Proper regulation of ROS levels is critical for plant growth and development (Liu et al., 2019; Tavanti et al., 2021). Plants typically counteract oxidative stress by synthesizing antioxidant enzymes such as superoxide dismutases and ascorbate peroxidases, and by producing antioxidant molecules including glutathione (GSH) and ascorbic acid (Martínez-Lorente et al., 2022). In addition, plants adopt other strategies to combat ROS accumulation. For instance, recent studies have shown that the chloroplast translocon complex (TOC) undergoes degradation under stress conditions, thereby reducing the import of photosynthetic components, suppressing photosynthesis, and ultimately limiting ROS accumulation (Ling et al., 2015). Our findings reveal that hydroxylated HPPD promotes binding to the E3 ubiquitin ligase PUB11, representing a novel mechanism that is essential for plant responses to oxidative stress.

HPPD is a critical enzyme in the biosynthesis of several key metabolites, including vitamin E, carotenoids, and plastoquinone. Because these metabolites have critical roles in photosynthesis and ROS scavenging, increased HPPD expression in response to stress-related stimuli such as salt, ethylene glycol, and abscisic acid (ABA) supports oxidative stress tolerance (Jiang et al., 2017; Kim et al., 2021; Fu et al., 2022; Lin et al., 2023). However, the detailed mechanisms through which HPPD responds to abiotic stress remain unclear. In this study, we explored the key processes underlying HPPD's response to oxidative stress. Comprehensive biochemical and genetic data strongly support our conclusions. First, *At*HPPD undergoes phenylalanine hydroxylation, a PTM that is significantly increased under oxidative stress conditions (Figure 1; Supplemental Figures 2 and 3). Second, *in vivo* and *in vitro* biochemical experiments revealed that phenylalanine hydroxylation affects *At*HPPD enzymatic activity, thereby facilitating its targeted degradation (Figure 2; Supplemental Figure 5; Table 1). Third, PUB11 interacts with *At*HPPD and promotes its degradation (Figures 3 and 4; Supplemental Figure 8). These findings demonstrate the critical role of HPPD in responding to oxidative stress and show that plants use diverse, multi-layered regulatory mechanisms to manage abiotic stress.

PTMs, such as oxidative modifications, are key regulators of protein function and are essential in the rapid response of plants to adversity (Lee et al., 2023). In *Arabidopsis*, GSTs (GSTF9 and GSTT23) exhibit a significant decrease in enzymatic activity following methionine oxidation (Jacques et al., 2015). Previous studies have demonstrated that *Pseudomonas* HPPD undergoes self-hydroxylation (Bradley et al., 1986; Liu et al., 2001). However, the physiological significance of HPPD hydroxylation is unclear, and it has not previously been observed in plant HPPD. In this study, we reveal that *At*HPPD undergoes hydroxylation at specific phenylalanine residues in response to oxidative stress (Figure 1A; Supplemental Figures 2 and 3). Under normal conditions, hydroxylation sites such as F419 display comparable modification levels in both *in vivo* and *in vitro* assays (Supplemental Figure 3D). However, under oxidative stress, most sites show a positive correlation with stress levels (Figure 1C). This study demonstrates that phenylalanine hydroxylation of HPPD occurs during the oxidative stress response, revealing a role for HPPD in stress mitigation through ROS regulation. In addition, as a critical component in photosynthesis, HPPD abundance is precisely regulated to maintain plant growth and development. Oxidative damage can inactivate HPPD (Supplemental Table 2); our results suggest that oxidized HPPD is targeted for degradation by the ubiquitin-proteasome system (UPS), representing a mechanism to recognize and remove dysfunctional proteins (Figures 4A and 4B; Supplemental Figure 5; Table 1). During photosynthesis, ROS are continuously produced and act as signaling molecules, potentially regulating HPPD protein levels by triggering its degradation through hydroxylation. This mechanism represents a straightforward but effective regulatory strategy that uses rapid protein-level modifications to adapt to fluctuating environmental conditions.

Numerous plant signaling pathways regulate the degradation of specific proteins via the ubiquitin–proteasome system (Trujillo, 2021; Trenner et al., 2022). Our results confirm that the degradation of *At*HPPD, essential for maintaining cellular function

under oxidative stress, is regulated by the 26S proteasome (Figures 4A–4C). Interaction assays demonstrated that PUB11 and *At*HPPD interact (Figure 3; Supplemental Table 3). Moreover, co-localization and BiFC results suggest that PUB11 mediates *At*HPPD degradation in the nucleus (Figure 3E; Supplemental Figure 11), consistent with previous studies showing that degradation of cytosolic proteins can occur in the nucleus (Shanmugabalaji et al., 2018). PUB11 was also confirmed as a key modulator of *At*HPPD degradation in both cell-free and *in vivo* degradation assays (Figures 4A–4C). *At*HPPD degradation occurred significantly faster in WT plants than in the *pub11* mutant (Figure 4F). Notably, the presence of PUB11 protein in the *pub11* mutant substantially increased the degradation rate of *At*HPPD (Figure 4E; Supplemental Figure 15). The enhanced binding affinity of hydroxylated *At*HPPD for PUB11 implies that hydroxylation promotes substrate degradation by facilitating this interaction (Figure 3D; Supplemental Figure 9). ROS levels are regulated by intricate networks of metabolic and signaling pathways. Under oxidative stress, ROS accumulation in PUB11-OE plants was significantly lower than in the WT and *pub11* mutant lines (Figure 4I). These results suggest that PUB11 contributes to ROS homeostasis, potentially by modulating HPPD stability. Overall, these findings suggest that hydroxylation of *At*HPPD phenylalanine residues under oxidative stress leads to the accumulation of inactive enzymes *in planta*. PUB11 may then facilitate the clearance of these inactive molecules, enabling renewed synthesis of active HPPD and increased tocopherol production to counter oxidative damage.

In summary, the present study uncovers a mechanism underlying *At*HPPD's response to oxidative stress, and we propose a putative working model of HPPD-mediated oxidative stress regulation in *Arabidopsis* (Figure 5). Excessive ROS trigger phenylalanine hydroxylation of *At*HPPD, compromising its enzymatic activity and leading to protein destabilization and proteasomal degradation. To safeguard normal growth and development, *Arabidopsis* uses the U-box E3 ubiquitin ligase PUB11 to ubiquitinate inactivated HPPD, triggering targeted 26S proteasome–mediated degradation. This clearance may more effectively promote the synthesis of active HPPD, thereby producing tocopherol to mitigate oxidative stress. Our model reveals a novel regulatory mechanism by which HPPD responds to oxidative stress. This discovery opens avenues for identifying innovative herbicide targets and establishes a foundation for enhancing crop resilience to environmental stressors.

METHODS

Plant materials and growth conditions

Arabidopsis plants used in this study were in the Col-0 background unless otherwise specified. The T-DNA insertion mutants were in the *pub11* background. To create overexpression lines, the full-length coding sequence of *At*HPPD was fused upstream of GFP in a pBWA(V)HS-GFP vector to generate the GFP-*At*HPPD-OE construct. An error-free clone was introduced into *Agrobacterium* strain GV3101 (Weidi Bioscience) and transformed into Col-0 using the standard floral-dip method. *At*HPPD expression levels were tested in T2 single-insertion homozygous lines, and homozygous T3 lines were used for further study. The primers used are listed in Supplemental Table 4.

Col-0 seeds were surface sterilized and incubated for 2 days at 4°C, then germinated and grown on 1/2 murashige and skoog medium (1/2 MS) (PhytoTech) with 1% sucrose and 0.8% (w/v) agar (BioFRox). At 5–7 days after germination, seedlings were transferred to soil and grown under a 16 h light/8 h dark photoperiod at 23°C. Seedlings with expanded cotyledons were considered green.

WT *N. benthamiana* seeds were grown in a growth chamber at 22°C under a 16 h light/8 h dark photoperiod with a light intensity of 400 $\mu mol\ m^{-2}\ s^{-1}$. After 4 weeks, the plants were used for *Agrobacterium*-mediated BiFC assays.

Y2H assays

The full-length PUB11 coding sequence (CDS) was cloned into the pGADT7 (prey) vector, and the full-length *At*HPPD CDS was cloned into the pGBKT7 (bait) vector. The bait and prey plasmids were co-transformed into the yeast strain Y2HGold (Coolaber). After growth in synthetic dropout (SD) solid medium lacking Trp and Leu (SD/−Trp/−Leu) (Coolaber) at 30°C for 3–4 days, the yeast cultures were spotted onto SD solid medium lacking Trp, His, and Leu but containing X-α-galactosidase (SD/−Trp/−His/−Leu/− + X-α-gal) (Coolaber) to test for growth and α-gal activity. Two independent clones were tested for each bait/prey combination.

In vitro pull-down assays

Pull-down assays were performed as described by Yang et al. (2008). The PUB11 CDS and three truncated variants were separately subcloned into the *pMAL-C2x* vector to add an MBP tag at the N terminus. The *At*HPPD CDS was subcloned into the *pET-15b*(+) vector to add an N-terminal 6×His tag. The resulting plasmids were introduced into *Escherichia coli* BL21(DE3) cells (AlpalifeBio). Expression of His-tagged and MBP-tagged fusion proteins was induced with 0.5 mM isopropyl-β-D-thiogalactoside at 18°C for 16 h. The recombinant proteins were purified and quantified according to the manufacturer's instructions (Novagen). Purified full-length MBP-PUB11, MBP-PUB11-UND, MBP-PUB11-U-box, MBP-PUB11-ARM, and His-*At*HPPD fusion proteins were subjected to *in vitro* pull-down assays at 4°C. SDS–PAGE and immunoblotting were then performed on the input and pull-down samples using an anti-His antibody (Zen-Bio) and an anti-MBP antibody (Proteintech).

BiFC assays

Using pEGOEP-35S vectors, full-length PUB11 and *At*HPPD were fused to the C-terminal or N-terminal fragments of YFP under the control of the cauliflower mosaic virus 35S promoter to generate PUB11-cYFP and *At*HPPD-nYFP, respectively. These constructs were co-transformed into 4-week-old *N. benthamiana* leaves using the *A. tumefaciens* method (Hiei et al., 1994). After 2–3 days, the YFP signal was observed under a confocal microscope (AXTi2-E, Nikon).

Subcellular co-localization

The PUB11 CDS was cloned into the pEGOEP-35S vector to fuse the enhanced green fluorescent protein CDS to the 3′ end of the PUB11 sequence. The *At*HPPD CDS was cloned into the pEGOEP-35S vector to fuse the mCherry CDS to the 3′ end of the *At*HPPD sequence. The constructs were transiently co-expressed in *Arabidopsis* protoplast cells as previously described (Yoo et al., 2007). Fluorescence was observed overnight using a confocal spectral microscope imaging system (NV01023, GE Healthcare).

IP–LC–MS/MS assay

To identify proteins that interact with *At*HPPD, we performed an IP–LC–MS/MS assay using 35S:*At*HPPD-GFP and 35S:GFP transgenic lines. Samples were collected and ground in five-fold (w/v) IP buffer (250 mM Tris–HCl [pH 7.5], 150 mM NaCl, 1 mM EDTA, 25% glycerol, 1× protease inhibitor cocktail cOmplete mini tablets [Thermo Scientific], 1% Triton X-100, and 50 μM MG132 [MCE]) using a grinder (Shanghai Jingxin Industrial Development). After collection on a magnetic rack at 4°C, GFP-agarose was collected and washed three times with IP wash

buffer (50 mM Tris–HCl [pH 7.5], 150 mM NaCl, and 1 mM EDTA). A volume of 50 μl elution buffer (200 mM glycine [pH 2.5]) was added to GFP-agarose, incubated at 4°C for 7 min, and repeated. The elution samples (about 100 μl in total) were neutralized by adding 1 M Tris–HCl (pH 10.4). Samples were collected and concentrated with an ultrafiltration column (Millipore) to a final volume of 50 μl in 50 mM Tris–HCl (pH 8.0) with 6 M guanidine hydrochloride. Protein concentrations in each fraction were quantified by a BCA assay, and the samples were stored at 4°C until use.

A total of 200 μg of each protein sample was digested using a 10 K filter as previously described (Wiśniewski et al., 2009). In brief, the sample was reduced with 20 mM dithiothreitol (DTT; Sigma), centrifuged for 20 min at 14 000*g* to remove the buffer and excess DTT, then alkylated with 20 mM iodoacetamide (Sigma) in Tris–HCl buffer (pH 8.2) in the dark for 30 min. After alkylation, the sample was washed three times with 50 mM NH_4HCO_3 by centrifugation at 14 000*g* for 20 min. Finally, trypsin (Promega) was added at an enzyme-to-protein mass ratio of 1:50; the digestion was carried out in 50 mM NH_4HCO_3. After overnight incubation at 37°C, the eluted peptides were collected by centrifugation at 14 000 *g* for 20 min.

The LC–MS/MS analysis was performed as previously described (Sun et al., 2019) with modifications. The peptide sample was loaded onto a reversed-phase μ-precolumn (particle size: 3 μm; Dionex/Thermo Scientific), and separation was performed using an analytical column (C18; particle size: 2 μm; Thermo Scientific, Germany) with a spray emitter for nano-electrospray ionization on a Q Exactive mass spectrometer (Thermo Scientific, USA) at a flow rate of 0.3 μl/min. Mobile phase A was 0.1% formic acid in H_2O, and mobile phase B was 80% acetonitrile, 19.9% H_2O, and 0.1% formic acid. Gradient elution was used to improve separation, using the following gradient: 0–4 min, 4%–8% B; 4–7 min, 8%–10% B; 7–52 min, 10%–25% B; 52–90 min, 25%–32% B; 90–95 min, 32%–40% B; 95–97 min, 40%–100% B; and 97–100 min, 100% B.

Intact peptides and ion fragments were detected in an Orbitrap mass spectrometer at resolutions of 70 000 and 17 500, respectively. A full mass spectrometry (MS) scan was acquired from *m/z* 350 to 1800. In data-dependent mode, the 20 most abundant ions were selected for MS/MS, using an automatic gain control target of 3×10^6, a normalized collision energy of 29%, dynamic exclusion set at 35.0 s, and electrospray voltage at 2.2 kV. All experiments were performed in triplicate.

The resulting MS/MS data were processed with Proteome Discoverer (v.3.0) (Thermo Scientific), using Sequest HT as the search engine. Tandem mass spectra were searched against a homemade *A. thaliana* database concatenated with a reverse decoy database. The homemade database contained 136 334 sequences. The false discovery rate for peptide identifications was controlled to less than 1%. For digested peptides, trypsin was assigned as the cleavage enzyme, allowing up to two missed cleavages. The mass error tolerance was set to 10 ppm for precursor ions and 0.02 Da for fragment ions. The minimum and maximum peptide lengths were set to 6 and 144 residues, respectively. Carbamidomethylation on cysteine (+57.021 Da) was set as a static modification, whereas methionine oxidation (+15.995 Da) and protein N-terminal acetylation (+42.011 Da) were set as dynamic modifications.

Protein degradation assay

A cell-free protein degradation assay was performed as previously described (Wang et al., 2009) with modifications. Total proteins from WT, *At*HPPD-GFP-OE, and *pub11* transgenic *A. thaliana* lines were extracted with degradation buffer (25 mM Tris–HCl [pH 7.5], 10 mM NaCl, 10 mM $MgCl_2$, 2 mM ATP, 5 mM DTT, and 1× protease inhibitor cocktail cOmplete mini tablets). Each reaction contained 500 μg of *A. thaliana* total protein and 100 ng of His-*At*HPPD protein. For the H_2O_2 stability assay, *At*HPPD was treated with 0, 50, or 250 mM H_2O_2. The reactions were incubated at 25°C and analyzed at time points from 0 to 2 h using an anti-His antibody (Cell Signaling).

For the proteasome inhibitor assay, 50 μM MG132 was added to total proteins from soybean 1 h before the cell-free degradation assay. The reactions were incubated at 25°C and analyzed at time points from 0 to 3 h, then terminated with 6 μl 6×SDS sample buffer at each time point. Samples were kept on ice until all reactions were complete, then incubated at 95°C for 8 min and subjected to western blot analysis with an anti-His antibody (Cell Signaling).

For Figures 2C and 2E, 10-day-old homozygous *At*HPPD-GFP-OE seedlings were treated with 100 μM CHX (MCE), with or without H_2O_2, for the specified durations. Seedlings were harvested and homogenized in liquid nitrogen. Proteins were extracted, mixed with 6×SDS loading buffer, heated at 95°C for 8 min, and centrifuged at room temperature for 3 min. The supernatants were analyzed by western blotting using an anti-GFP antibody (Proteintech).

For Figure 4A, supernatants of extracts from *At*HPPD-GFP-OE seedlings were divided into aliquots and incubated with or without 2 mM ATP at 25°C for different durations. Reactions were stopped using 6×SDS loading buffer, then the samples were boiled and analyzed by immunoblotting with an anti-GFP antibody (Proteintech).

For Figures 4D and 4E, supernatants of extracts from WT or *pub11* plants were divided into aliquots and incubated with or without 2 μg PUB11 protein at 25°C for different durations. Reactions were stopped using 6×SDS loading buffer, then the samples were boiled and analyzed by immunoblotting with an anti-His antibody (Cell Signaling).

In vivo ubiquitination assays

The *in vivo* ubiquitination of *At*HPPD in protoplasts was performed as described by Wan et al. (2023) with modifications. In brief, *At*HPPD-GFP-OE seedlings were grown under a 16 h light/8 h dark photoperiod at 22°C on plates for about 21 days. For IP assays, 1 ml of *Arabidopsis* protoplasts (10^6 cells) was transfected with 100 μg of DNA. After 18 h, the protoplasts were treated with 5 μM MG132 for 4 h, followed by extraction. Pellets were lysed in 200 μl degradation buffer (2 mM MES–KOH [pH 5.6], 150 mM NaCl, 125 mM $CaCl_2$, and 5 mM KCl). The supernatants were incubated overnight at 4°C with 30 μl beads coated with an anti-GFP antibody (Proteintech). After three washes with IP buffer (250 mM Tris–HCl [pH 7.5], 300 mM NaCl, 2 mM EDTA, 25% glycerol, 1× protease inhibitor cocktail cOmplete mini tablets, 1% Triton X-100, and 50 μM MG132), proteins were eluted with SDS loading buffer and immunoblotted using anti-GFP (Proteintech), anti-Myc (Sigma), anti-FLAG (Proteintech), and anti-actin (HUABIO) antibodies.

In vivo protein degradation

In vivo degradation of *At*HPPD was performed in protoplasts as described by Wan et al. (2023) with minor modifications. Protoplast preparation was performed as described for the ubiquitination assay. The experimental group involved transient expression of 35S:*PUB11-MYC*, whereas the control group lacked PUB11 expression. CHX (5 μM) was added 12 h post-transfection, and samples were collected 4 h later for analysis. *At*HPPD was enriched using GFP beads and analyzed by immunoblotting.

Protein expression and purification

Full-length *At*HPPD in the *pET-15b* vector was expressed and purified as described in Yan et al. (2022). The full-length PUB11 coding sequence was cloned into the *pMAL-C2X* vector, and the PUB11 deletion variants were cloned into either the *pMAL-C2X* or the *pCool* vector. The plasmids were transformed into *E. coli* BL21(DE3). One liter of lysogeny broth medium supplemented with 100 μg ml^{-1} ampicillin was inoculated with a transformed bacterial preculture and shaken at 37°C until the cell density reached an OD_{600} of 0.8–1.0. Protein expression was induced with 0.2 mM

isopropyl-β-D-thiogalactoside at 20°C for 12–16 h, then cells were collected by centrifugation. The PUB11 full-length and deletion variants expressed from the pMAL-C2X vector were homogenized in buffer A (20 mM Tris–HCl [pH 7.4] and 200 mM NaCl). Cell debris was removed by centrifugation at 14 000*g* and 4°C for 1 h, then the supernatant was loaded onto a column with MBP affinity resin (YEASEN), washed with buffer B (20 mM Tris–HCl [pH 7.4], 200 mM NaCl, and 1 mM EDTA), and eluted with buffer C (20 mM Tris–HCl [pH 7.4], 1 mM EDTA, and 10 mM maltose). PUB11 expressed from the *pCool vector was* homogenized in buffer A (20 mM Tris–HCl [pH 8.0] and 200 mM NaCl). Cell debris was removed by centrifugation at 14 000*g* and 4°C for 1 h, then the supernatant was loaded onto a column with GST affinity resin (GE), washed with buffer B (20 mM Tris–HCl [pH 8.0] and 500 mM NaCl), and eluted with buffer C (10 mM GSH and 20 mM Tris–HCl [pH 8.5]). The protein concentration was determined using a bicinchoninic acid assay kit (Biosharp), and proteins were stored at −80°C.

HPPD activity assay

The coupled enzyme assay for *in vitro* activity was performed as previously described (Lin et al., 2019; Yu et al., 2023) with modifications. The reaction mixture contained 20 mM HEPES (pH 7.0), 2 mM sodium ascorbate, 100 μM $FeSO_4$, and a series of HPPA concentrations (200, 100, 70, 50, 30, 20, 15, 10, 7, 5, and 2 μM), along with sufficient homogentisate 1,2-dioxygenase. The mixture was incubated for approximately 15 min; the reaction was then initiated by adding HPPD, and the absorbance change was measured at 318 nm. The production of maleylacetoacetate was quantified spectrophotometrically using a Molecular Devices Synergy H1 microplate reader (BioTek, Winooski, VT). The derivations of the related K_m and k_{cat} formulas have been reported previously (Lin et al., 2021).

Quantification of *At*HPPD phenylalanine hydroxylation

To quantify phenylalanine hydroxylation abundance, *At*HPPD was treated with 0, 50, or 250 mM H_2O_2, followed by digestion with trypsin. Tryptic peptides were analyzed by LC–MS/MS as described previously (Sun et al., 2019). Peptides were injected into an Ultimate 3000 RSLC Nano System (Dionex, USA) coupled to an Orbitrap Exploris 480 mass spectrometer (Thermo Scientific). The peptides were loaded onto a C18 PepMap100 precolumn (particle size: 3 μm; Dionex/Thermo Scientific) and an analytical column (C18; particle size: 2 μm; Thermo Scientific, Germany) using a 50 min gradient with high-performance LC (HPLC) buffer A (0.1% formic acid/H_2O) and buffer B (80% acetonitrile, 19.9% H_2O, and 0.1% formic acid). Gradient elution was used to improve separation, using the following gradient: 0–3 min, 6%–10% B; 3–42 min, 10%–32% B; 42–46 min, 32%–94% B; and 46–50 min, 94% B. A label-free approach was used to monitor precursor ions and identify specific HPLC elution peaks. The peak areas of targeted ions in the HPLC chromatogram were calculated using Proteome Discoverer (v.3.0) to determine the abundance of modified peptides. Stoichiometry was calculated as previously described (Erber et al., 2019): %Hyp = (peak area of Hyp-containing peptide)/(peak area of Hyp-containing peptide + peak area of unmodified peptide) × 100. Three biological replicates were analyzed under each condition, and statistical significance was assessed using a two-tailed unpaired Student's *t*-test.

MST assays

The MST assay was performed as previously described (Wienken et al., 2010; Jerabek-Willemsen et al., 2014). His-*At*HPPD and its mutants were labeled with red fluorescent dye using the His-Tag Labeling Kit RED-tris-NTA 2nd generation (NanoTemper Technologies, München, Germany). In *At*HPPD/PUB11-ARM interaction assays, the concentration of His-labeled *At*HPPD was kept constant at 400 nM, whereas PUB11-ARM was serially diluted. Measurements were performed at 25°C in a buffer containing 20 mM HEPES (pH 8.0) and 100 mM NaCl. Each affinity measurement was repeated three times. Data analyses were performed using NanoTemper Analysis (NanoTemper Technologies) and OriginPro 8.0 (OriginLab). For interaction assays, GST-PUB11-ARM or GST was added to His-labeled *At*HPPD. After a 30-min incubation, the samples were loaded into MST standard-treated glass capillaries for MST analysis as described above.

Quantitative RT–PCR analysis

Extraction of *Arabidopsis* RNA and quantitative RT–PCR were performed using established methods (Jozefczuk et al., 2011; Ma et al., 2021). In brief, quantitative RT–PCR analysis was performed using the ABI QuantStudio 1 Real-Time PCR System (Thermo Scientific) and PerfectStart Green qPCR SuperMix (TransGen Biotech). Gene-specific primers were pub11-F and pub11-R for PUB11, GFP-*At*HPPD-F and GFP-*At*HPPD-R for *At*HPPD, and actin-F and actin-R for the actin internal control. The specific primers are listed in Supplemental Table 4.

Stress treatment and detection of plant ROS

Ten-day-old seedlings of PUB11-OE, *pub11,* and WT plants were treated with 50 μM H_2O_2 for 36 h in the greenhouse as described above. H_2O_2 accumulation was measured using a plant ROS enzyme-linked immunosorbent assay kit (shrjbio).

Circular dichroism spectroscopy

Circular dichroism experiments were performed using a Jasco J-1500 spectropolarimeter (Tokyo, Japan). Ellipticity in the UV region was measured at room temperature in 10 mM phosphate buffer (pH 7.0) using a quartz cuvette with a 1 mm pathlength.

Extraction and quantitative analysis of tocopherol

For tocopherol analysis, green leaf tissue was harvested from 20-day-old *Arabidopsis* seedlings (50 mg) and immediately frozen in liquid nitrogen. The samples were ground in 1 ml of methanol/chloroform (2:1, v/v) containing 0.01% butylated hydroxytoluene (Aladdin), and the homogenized tissue was incubated for 20 min at room temperature. After the addition of 300 μl chloroform (SINOPHARM) and 600 μl H_2O, the mixture was vortexed thoroughly and centrifuged at 14 000*g* for 10 min at room temperature to separate the phases. The tocopherol-containing chloroform phase was collected and dried under vacuum to remove the solvent. Dried samples were resuspended in 400 μl of a 1:5 (v/v) mixture of dichloromethane (Energy Chemical) and methanol (Thermo Scientific), and a 35 μl aliquot was injected into an Agilent-C18 column (4.6 × 250 mm, 5 μm particle size) using a mobile phase of methanol and water (98:2, v/v) at a flow rate of 1.5 ml/min. The column temperature was maintained at 30°C. Sample components were detected and quantified by fluorescence with excitation at 290 nm and emission at 330 nm.

Statistical analysis

All statistical analyses were performed in IBM SPSS v.26 (SPSS, Chicago, IL, USA) using Fisher's least significant difference test. No statistical methods were used to predetermine the sample size. No data were excluded from the analysis. Samples were grown under uniform conditions and randomly allocated within the growth chamber. Experimental plant material was collected randomly to avoid bias. Investigators were not blinded to allocation during the experiments or outcome assessment.

DATA AND CODE AVAILABILITY

All data are available in the main text or the supplemental information.

FUNDING

We are grateful to the National Natural Science Foundation of China (22377031), the China Postdoctoral Science Foundation under grant number 2024M761101, the Strategic Priority Research Program of the Chinese Academy of Sciences (XDB0630000), the Hubei Provincial Science and Technology Plan Project (2022BEC051), self-determined research funds of CCNU from the Colleges' Basic Research and Operation of MOE (CCNU24JCPT023), and the Key Project of the Natural Science Foundation of Hubei Province (2025AFA078).

ACKNOWLEDGMENTS
No conflict of interest declared.

AUTHOR CONTRIBUTIONS
G.-F.Y., H.-Y.L., and Q.L. supervised the project. X.-H.Y., X.W., and J.D. designed the research. X.-H.Y. and J.D. wrote the paper. X.-H.Y., X.W., and Y.-F.H. performed most of the experiments. X.-L.W. and D.-Y.Z. provided technical assistance. X.-H.Y., X.W., J.D., H.-Y.L., Q.L., and G.-F.Y. discussed the study and revised the manuscript. H.-Y.L., Q.L., and X.-H.Y. secured the necessary funding.

SUPPLEMENTAL INFORMATION
Supplemental information is available at *Plant Communications Online*.

Received: June 15, 2025
Revised: August 16, 2025
Accepted: September 5, 2025
Published: September 8, 2025

REFERENCES
Bradley, F.C., Lindstedt, S., Lipscomb, J.D., Que, L., Roe, A.L., and Rundgren, M. (1986). 4-Hydroxyphenylpyruvate dioxygenase is an iron-tyrosinate protein. J. Biol. Chem. **261**:11693–11696. https://doi.org/10.1016/S0021-9258(18)67299-0.

Chen, X., Wang, T., Rehman, A.U., Wang, Y., Qi, J., Li, Z., Song, C., Wang, B., Yang, S., and Gong, Z. (2021). Arabidopsis U-box E3 ubiquitin ligase PUB11 negatively regulates drought tolerance by degrading the receptor-like protein kinases LRR1 and KIN7. J. Integr. Plant Biol. **63**:494–509. https://doi.org/10.1111/jipb.13058.

Erber, L., Luo, A., and Chen, Y. (2019). Targeted and Interactome Proteomics Revealed the Role of PHD2 in Regulating BRD4 Proline Hydroxylation. Mol. Cell. Proteomics **18**:1772–1781. https://doi.org/10.1074/mcp.RA119.001535.

Fong, G.-H., and Takeda, K. (2008). Role and regulation of prolyl hydroxylase domain proteins. Cell Death Differ. **15**:635–641. https://doi.org/10.1038/cdd.2008.10.

Fu, Y.X., Liu, S.Y., Guo, W.Y., Dong, J., Nan, J.X., Lin, H.Y., Mei, L.C., Yang, W.C., and Yang, G.F. (2022). In vivo diagnostics of abiotic plant stress responses via in situ real-time fluorescence imaging. Plant Physiol. **190**:196–201. https://doi.org/10.1093/plphys/kiac273.

Guccione, E., and Richard, S. (2019). The regulation, functions and clinical relevance of arginine methylation. Nat. Rev. Mol. Cell Biol. **20**:642–657. https://doi.org/10.1038/s41580-019-0155-x.

Han, R., Ma, L., Lv, Y., Qi, L., Peng, J., Li, H., Zhou, Y., Song, P., Duan, J., Li, J., et al. (2023). SALT OVERLY SENSITIVE2 stabilizes phytochrome-interacting factors PIF4 and PIF5 to promote Arabidopsis shade avoidance. Plant Cell **35**:2972–2996. https://doi.org/10.1093/plcell/koad119.

Hiei, Y., Ohta, S., Komari, T., and Kumashiro, T. (1994). Efficient transformation of rice (Oryza sativa L.) mediated by Agrobacterium and sequence analysis of the boundaries of the T-DNA. Plant J. **6**:271–282. https://doi.org/10.1046/j.1365-313x.1994.6020271.x.

Islam, M.S., Leissing, T.M., Chowdhury, R., Hopkinson, R.J., and Schofield, C.J. (2018). 2-Oxoglutarate-Dependent Oxygenases. Annu. Rev. Biochem. **87**:585–620. https://doi.org/10.1146/annurev-biochem-061516-044724.

Ivan, M., Kondo, K., Yang, H., Kim, W., Valiando, J., Ohh, M., Salic, A., Asara, J.M., Lane, W.S., and Kaelin, W.G. (2001). HIFalpha targeted for VHL-mediated destruction by proline hydroxylation: implications for O2 sensing. Science **292**:464–468. https://doi.org/10.1126/science.1059817.

Jacques, S., Ghesquière, B., De Bock, P.J., Demol, H., Wahni, K., Willems, P., Messens, J., Van Breusegem, F., and Gevaert, K. (2015). Protein Methionine Sulfoxide Dynamics in Arabidopsis thaliana under Oxidative Stress. Mol. Cell. Proteomics **14**:1217–1229. https://doi.org/10.1074/mcp.M114.043729.

Jerabek-Willemsen, M., André, T., Wanner, R., Roth, H.M., Duhr, S., Baaske, P., and Breitsprecher, D. (2014). MicroScale Thermophoresis: Interaction analysis and beyond. J. Mol. Struct. **1077**:101–113. https://doi.org/10.1016/j.molstruc.2014.03.009.

Jiang, J., Chen, Z., Ban, L., Wu, Y., Huang, J., Chu, J., Fang, S., Wang, Z., Gao, H., and Wang, X. (2017). P-HYDROXYPHENYLPYRUVATE DIOXYGENASE from Medicago sativa is involved in vitamin E biosynthesis and abscisic acid-mediated seed germination. Sci. Rep. **7**:40625. https://doi.org/10.1038/srep40625.

Jozefczuk, J., and Adjaye, J. (2011). Chapter Six - Quantitative Real-Time PCR-Based Analysis of Gene Expression. Methods Enzymol. **500**:99–109. https://doi.org/10.1016/B978-0-12-385118-5.00006-2.

Kim, S.E., Bian, X., Lee, C.J., Park, S.U., Lim, Y.H., Kim, B.H., Park, W. S., Ahn, M.J., Ji, C.Y., Yu, Y., et al. (2021). Overexpression of 4-hydroxyphenylpyruvate dioxygenase (IbHPPD) increases abiotic stress tolerance in transgenic sweetpotato plants. Plant Physiol. Biochem. **167**:420–429. https://doi.org/10.1016/j.plaphy.2021.08.025.

Lee, J.M., Hammarén, H.M., Savitski, M.M., and Baek, S.H. (2023). Control of protein stability by post-translational modifications. Nat. Commun. **14**:201. https://doi.org/10.1038/s41467-023-35795-8.

Li, M., and Kim, C. (2022). Chloroplast ROS and stress signaling. Plant Commun. **3**:100264. https://doi.org/10.1016/j.xplc.2021.100264.

Li, P., and Liu, J. (2021). Protein Phosphorylation in Plant Cell Signaling. Methods Mol. Biol. **2358**:45–71. https://doi.org/10.1007/978-1-0716-1625-3_3.

Li, F., Zhang, F., Yi, X., Quan, L.L., Yang, X., Yin, C., Ma, Z., Wu, R., Zhao, W., Ling, M., et al. (2023). Proline hydroxylase 2 (PHD2) promotes brown adipose thermogenesis by enhancing the hydroxylation of UCP1. Mol. Metab. **73**:101747. https://doi.org/10.1016/j.molmet.2023.101747.

Lin, H.Y., Chen, X., Chen, J.N., Wang, D.W., Wu, F.X., Lin, S.Y., Zhan, C.G., Wu, J.W., Yang, W.C., and Yang, G.F. (2019). Crystal Structure of 4-Hydroxyphenylpyruvate Dioxygenase in Complex with Substrate Reveals a New Starting Point for Herbicide Discovery. Research (Wash D C) **2019**:2602414. https://doi.org/10.34133/2019/2602414.

Lin, H.Y., Chen, X., Dong, J., Yang, J.F., Xiao, H., Ye, Y., Li, L.H., Zhan, C.G., Yang, W.C., and Yang, G.F. (2021). Rational Redesign of Enzyme via the Combination of Quantum Mechanics/Molecular Mechanics, Molecular Dynamics, and Structural Biology Study. J. Am. Chem. Soc. **143**:15674–15687. https://doi.org/10.1021/jacs.1c06227.

Lin, H.Y., Dong, J., Dong, J., Yang, W.C., and Yang, G.F. (2023). Insights into 4-hydroxyphenylpyruvate dioxygenase-inhibitor interactions from comparative structural biology. Trends Biochem. Sci. **48**:568–584. https://doi.org/10.1016/j.tibs.2023.02.006.

Ling, Q., and Jarvis, P. (2015). Regulation of Chloroplast Protein Import by the Ubiquitin E3 Ligase SP1 Is Important for Stress Tolerance in Plants. Curr. Biol. **25**:2527–2534. https://doi.org/10.1016/j.cub.2015.08.015.

Ling, Q., Huang, W., Baldwin, A., and Jarvis, P. (2012). Chloroplast biogenesis is regulated by direct action of the ubiquitin-proteasome system. Science **338**:655–659. https://doi.org/10.1126/science.1225053.

Liu, A., Ho, R.Y., Que, L., Ryle, M.J., Phinney, B.S., and Hausinger, R.P. (2001). Alternative Reactivity of an α-Ketoglutarate-Dependent Iron(II) Oxygenase: Enzyme Self-Hydroxylation. J. Am. Chem. Soc. **123**:5126–5127. https://doi.org/10.1021/ja005879x.

Liu, C., Ma, H., Zhou, J., Li, Z., Peng, Z., Guo, F., and Zhang, J. (2019). TsHD1 and TsNAC1 cooperatively play roles in plant growth and

abiotic stress resistance of Thellungiella halophile. Plant J. **99**:81–97. https://doi.org/10.1111/tpj.14310.

Ma, H., Bell, K.N., and Loker, R.N. (2021). qPCR and qRT-PCR analysis: Regulatory points to consider when conducting biodistribution and vector shedding studies. Mol. Ther. Methods Clin. Dev. **20**:152–168. https://doi.org/10.1016/j.omtm.2020.11.007.

Mangano, S., Juárez, S.P.D., and Estevez, J.M. (2016). ROS Regulation of Polar Growth in Plant Cells. Plant Physiol. **171**:1593–1605. https://doi.org/10.1104/pp.16.00191.

Martínez-Lorente, S.E., Pardo-Hernández, M., Martí-Guillén, J.M., López-Delacalle, M., and Rivero, R.M. (2022). Interaction between Melatonin and NO: Action Mechanisms, Main Targets, and Putative Roles of the Emerging Molecule NOmela. Int. J. Mol. Sci. **23**:6646. https://doi.org/10.3390/ijms23126646.

Mekhail, K., Gunaratnam, L., Bonicalzi, M.E., and Lee, S. (2004). HIF activation by pH-dependent nucleolar sequestration of VHL. Nat. Cell Biol. **6**:642–647. https://doi.org/10.1038/ncb1144.

Ruiz-May, E., Segura-Cabrera, A., Elizalde-Contreras, J.M., Shannon, L.M., and Loyola-Vargas, V.M. (2019). A recent advance in the intracellular and extracellular redox post-translational modification of proteins in plants. J. Mol. Recognit. **32**:e2754. https://doi.org/10.1016/S0981-9428(02)01461-4.

Shanmugabalaji, V., Chahtane, H., Accossato, S., Rahire, M., Gouzerh, G., Lopez-Molina, L., and Kessler, F. (2018). Chloroplast Biogenesis Controlled by DELLA-TOC159 Interaction in Early Plant Development. Curr. Biol. **28**:2616–2623.e5. https://doi.org/10.1016/j.cub.2018.06.006.

Smalle, J., and Vierstra, R.D. (2004). The ubiquitin 26S proteasome proteolytic pathway. Annu. Rev. Plant Biol. **55**:555–590. https://doi.org/10.1146/annurev.arplant.55.031903.141801.

Sun, Z., Yu, X., Zhang, Y., Xu, J., and Li, X. (2019). Construction of a comprehensive beer proteome map using sequential filter-aided sample preparation coupled with liquid chromatography tandem mass spectrometry. J. Sep. Sci. **42**:2835–2841. https://doi.org/10.1002/jssc.201900074.

Tsegaye, Y., Shintani, D.K., and DellaPenna, D. (2002). Overexpression of the enzyme p-hydroxyphenolpyruvate dioxygenase in Arabidopsis and its relation to tocopherol biosynthesis. Plant Physiol. Biochem. **40**:913–920. https://doi.org/10.1016/S0981-9428(02)01461-4.

Tavanti, T.R., Melo, A.A.R.d., Moreira, L.D.K., Sanchez, D.E.J., Silva, R.D.S., Silva, R.M.d., and Reis, A.R.D. (2021). Micronutrient fertilization enhances ROS scavenging system for alleviation of abiotic stresses in plants. Plant Physiol. Biochem. **160**:386–396. https://doi.org/10.1016/j.plaphy.2021.01.040.

Trenner, J., Monaghan, J., Saeed, B., Quint, M., Shabek, N., and Trujillo, M. (2022). Evolution and Functions of Plant U-Box Proteins: From Protein Quality Control to Signaling. Annu. Rev. Plant Biol. **73**:93–121. https://doi.org/10.1146/annurev-arplant-102720-012310.

Trujillo, M. (2021). Ubiquitin signalling: controlling the message of surface immune receptors. New Phytol. **231**:47–53. https://doi.org/10.1111/nph.17360.

Wan, C., Zhang, H., Cheng, H., Sowden, R.G., Cai, W., Jarvis, R.P., and Ling, Q. (2023). Selective autophagy regulates chloroplast protein import and promotes plant stress tolerance. EMBO J. **42**:e112534. https://doi.org/10.15252/embj.2022112534.

Wang, F., Zhu, D., Huang, X., Li, S., Gong, Y., Yao, Q., Fu, X., Fan, L.-M., and Deng, X.W. (2009). Biochemical Insights on Degradation of Arabidopsis DELLA Proteins Gained From a Cell-Free Assay System. Plant Cell **21**:2378–2390. https://doi.org/10.1105/tpc.108.065433.

Wang, D.W., Lin, H.Y., Cao, R.J., Chen, T., Wu, F.X., Hao, G.F., Chen, Q., Yang, W.C., and Yang, G.F. (2015). Synthesis and Herbicidal Activity of Triketone–Quinoline Hybrids as Novel 4-Hydroxyphenylpyruvate Dioxygenase Inhibitors. J. Agric. Food Chem. **63**:5587–5596. https://doi.org/10.1021/acs.jafc.5b01530.

Wienken, C.J., Baaske, P., Rothbauer, U., Braun, D., and Duhr, S. (2010). Protein-binding assays in biological liquids using microscale thermophoresis. Nat. Commun. **1**:100. https://doi.org/10.1038/ncomms1093.

Wiśniewski, J.R., Zougman, A., Nagaraj, N., and Mann, M. (2009). Universal sample preparation method for proteome analysis. Nat. Methods **6**:359–362. https://doi.org/10.1038/nmeth.1322.

Xie, X., He, Z., Chen, N., Tang, Z., Wang, Q., and Cai, Y. (2019). The Roles of Environmental Factors in Regulation of Oxidative Stress in Plant. BioMed Res. Int. **2019**:9732325. https://doi.org/10.1155/2019/9732325.

Yan, Y.C., Wu, W., Huang, G.Y., Yang, W.C., Chen, Q., Qu, R.Y., Lin, H.Y., and Yang, G.F. (2022). Pharmacophore-Oriented Discovery of Novel 1,2,3-Benzotriazine-4-one Derivatives as Potent 4-Hydroxyphenylpyruvate Dioxygenase Inhibitors. J. Agric. Food Chem. **70**:6644–6657. https://doi.org/10.1021/acs.jafc.2c01507.

Yang, L., Jiang, Y., Wu, S.F., Zhou, M.Y., Wu, Y.L., and Chen, G.Q. (2008). CCAAT/enhancer-binding protein alpha antagonizes transcriptional activity of hypoxia-inducible factor 1 alpha with direct protein-protein interaction. Carcinogenesis **29**:291–298. https://doi.org/10.1093/carcin/bgm262.

Yoo, S.D., Cho, Y.H., and Sheen, J. (2007). Arabidopsis mesophyll protoplasts: a versatile cell system for transient gene expression analysis. Nat. Protoc. **2**:1565–1572. https://doi.org/10.1038/nprot.2007.199.

Yu, X.H., Dong, J., Fan, C.P., Chen, M.X., Li, M., Zheng, B.F., Hu, Y.F., Lin, H.Y., and Yang, G.F. (2023). Discovery and Development of 4-Hydroxyphenylpyruvate Dioxygenase as a Novel Crop Fungicide Target. J. Agric. Food Chem. **71**:19396–19407. https://doi.org/10.1021/acs.jafc.3c05260.

Zeng, X., Ma, X., Dong, J., Li, B., Hua Liu, S., Yin, J., and Yang, G.F. (2023). A Protocol for Activated Bioorthogonal Fluorescence Labeling and Imaging of 4-Hydroxyphenylpyruvate Dioxygenase in Plants. Angew. Chem. Int. Ed. Engl. **62**:e202312618. https://doi.org/10.1002/anie.202312618.

Zhang, W., Ames, B.D., and Walsh, C.T. (2011). Identification of phenylalanine 3-hydroxylase for meta-tyrosine biosynthesis. Biochemistry **50**:5401–5403. https://doi.org/10.1021/bi200733c.

Zhang, Y., Xu, J., Li, R., Ge, Y., Li, Y., and Li, R. (2023a). Plants' Response to Abiotic Stress: Mechanisms and Strategies. Int. J. Mol. Sci. **24**:10915. https://doi.org/10.3390/ijms241310915.

Zhang, X.Y., Tang, L.H., Nie, J.W., et al. (2023b). Structure and activation mechanism of the rice Salt Overly Sensitive 1 (SOS1) Na+/H+ antiporter. Nat. Plants **9**:1924–1936. https://doi.org/10.1038/s41477-023-01551-5.

Zhang, H., Zhu, J., Gong, Z., and Zhu, J.K. (2022). Abiotic stress responses in plants. Nat. Rev. Genet. **23**:104–119. https://doi.org/10.1038/s41576-021-00413-0.

Zhu, J.K. (2016). Abiotic Stress Signaling and Responses in Plants. Cell **167**:313–324. https://doi.org/10.1016/j.cell.2016.08.029.

Plant Communications, Volume *6*

Supplemental information

Hydroxylation of HPPD facilitates its PUB11-mediated ubiquitination and degradation in response to oxidative stress in *Arabidopsis*

Xin-He Yu, Xun Wen, Jiangqing Dong, Ya-Fang Hu, Xin-Long Wang, Dan-Yi Zhu, Qihua Ling, Hong-Yan Lin, and Guang-Fu Yang

Supporting information

Hydroxylation of HPPD facilitates its PUB11-mediated ubiquitination and degradation in response to oxidative stress in *Arabidopsis*

Xin-He Yu[1,2]†, Xun Wen[1,2]†, Jiangqing Dong[3,4]†, Ya-Fang Hu[1,2], Xin-Long Wang[1,2], Dan-Yi Zhu[1,2], Qihua Ling[5,6]*, Hong-Yan Lin[1,2]*, Guang-Fu Yang[1,2]*

[1]State Key Laboratory of Green Pesticide, Central China Normal University, Wuhan 430079, PR China.

[2]International Joint Research Center for Intelligent Biosensor Technology and Health, Central China Normal University, Wuhan 430079, PR China.

[3]Hubei Shizhen Laboratory, Wuhan 430061, PR China.

[4]School of Basic Medical Sciences, Hubei University of Chinese Medicine, Wuhan 430065, PR China.

[5]Key Laboratory of Plant Carbon Capture, CAS Centre for Excellence in Molecular Plant Sciences, Institute of Plant Physiology and Ecology, Chinese Academy of Sciences, Shanghai, China.

[6]CAS-JIC Center of Excellence for Plant and Microbial Sciences (CEPAMS), Institute of Plant Physiology and Ecology, Chinese Academy of Sciences, Shanghai, China.

***Corresponding author. Email: qhling@cemps.ac.cn;hylin@ccnu.edu.cn; gfyang@ccnu.edu.cn.**

†These authors contributed equally to this work.

The file includes:

Figs. S1 to S17

Tables S1 to S4

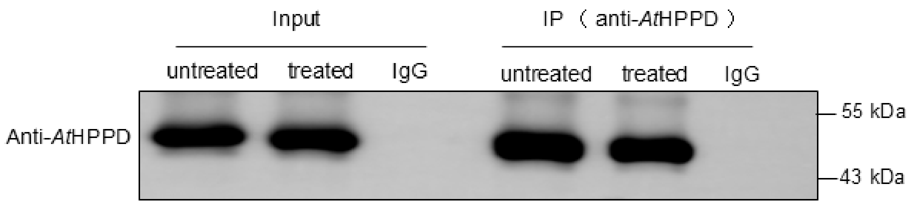

Fig. S1. *In vivo* enrichment of *At*HPPD in IP assays. Two-week-old WT plants were grown under normal growth condition, or subjected to H_2O_2 stress, with H_2O_2 treatment for 24 h. The protein extract from the plants was analyzed by IP using *At*HPPD antibody, or IgG as a negative control, and analyzed by immunoblotting using antibodies as indicated to the image.

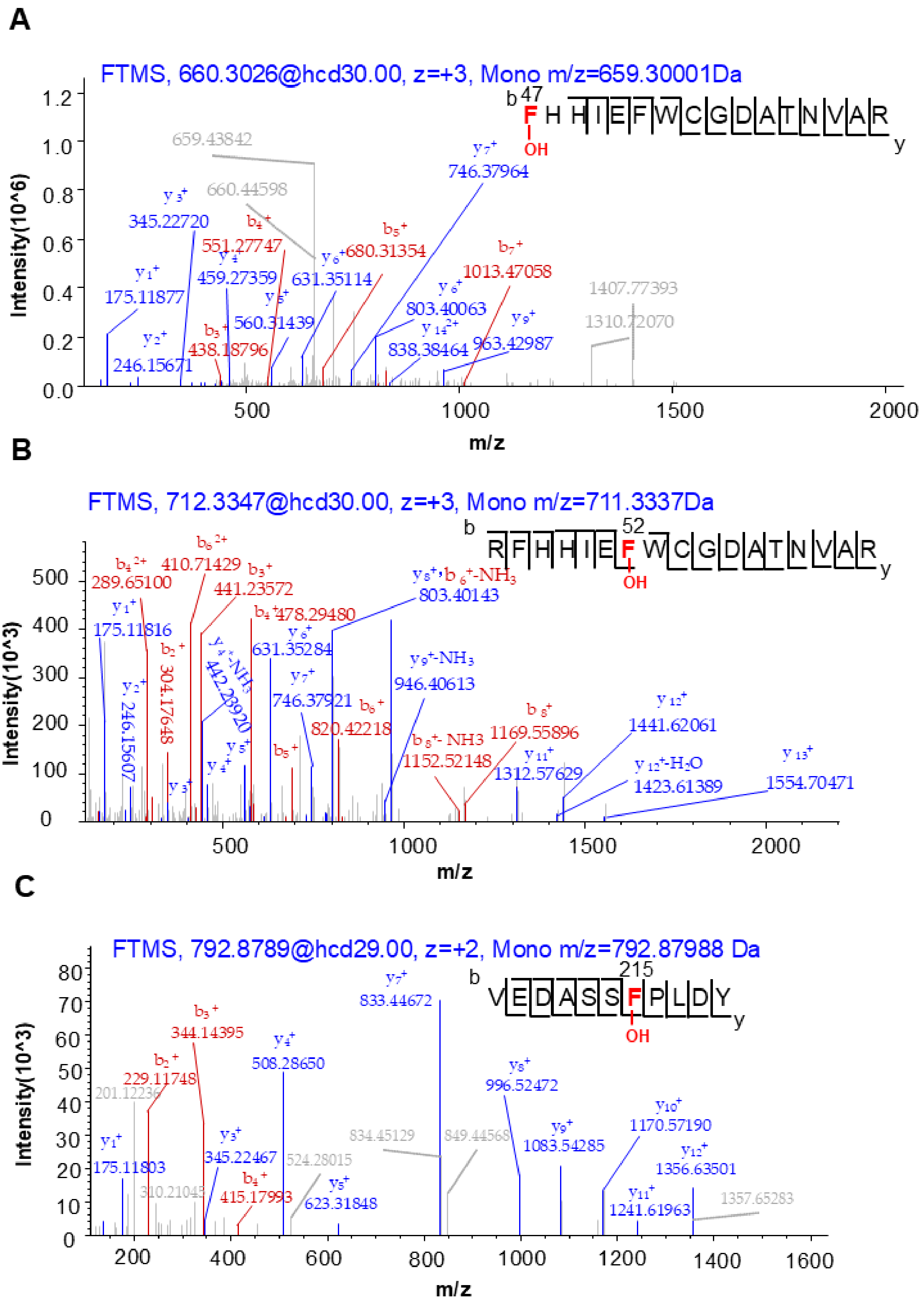
A
FTMS, 660.3026@hcd30.00, z=+3, Mono m/z=659.30001Da
b 47 F H H I E F W C G D A T N V A R y
OH
Intensity(10^6)
m/z
B
FTMS, 712.3347@hcd30.00, z=+3, Mono m/z=711.3337Da
b 52 R F H H I E F W C G D A T N V A R y
OH
Intensity(10^3)
m/z
C
FTMS, 792.8789@hcd29.00, z=+2, Mono m/z=792.87988 Da
b 215 V E D A S S F P L D Y y
OH
Intensity(10^3)
m/z

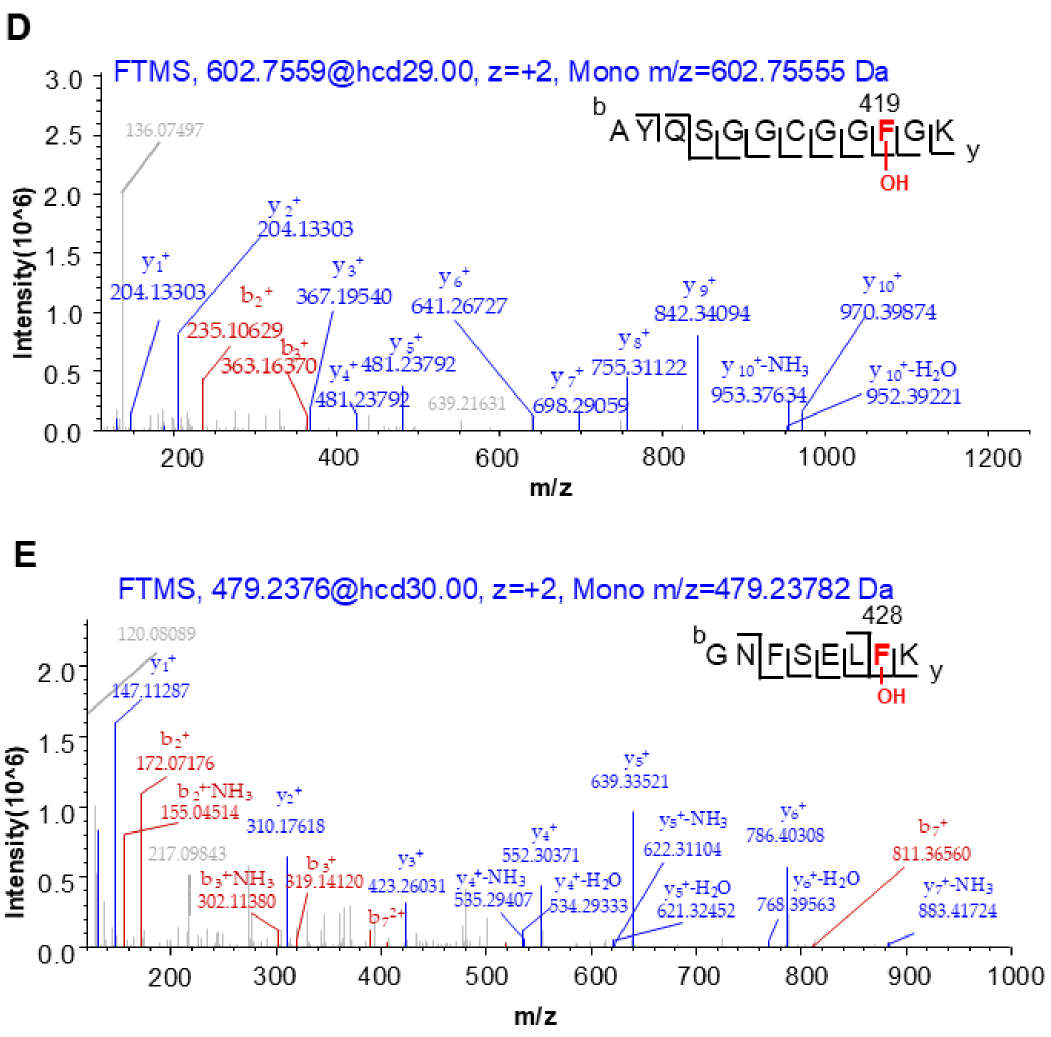

Fig. S2. Identification of HPPD modification sites *in vivo* by LC-MS/MS. (A) The product ion spectrum of the residue F47 of *At*HPPD. (B) The product ion spectrum of the residue F52 of *At*HPPD. (C) The product ion spectrum of the residue F215 of *At*HPPD. (D) The product ion spectrum of the residue F419 of *At*HPPD. (E) The product ion spectrum of the residue F428 of *At*HPPD.

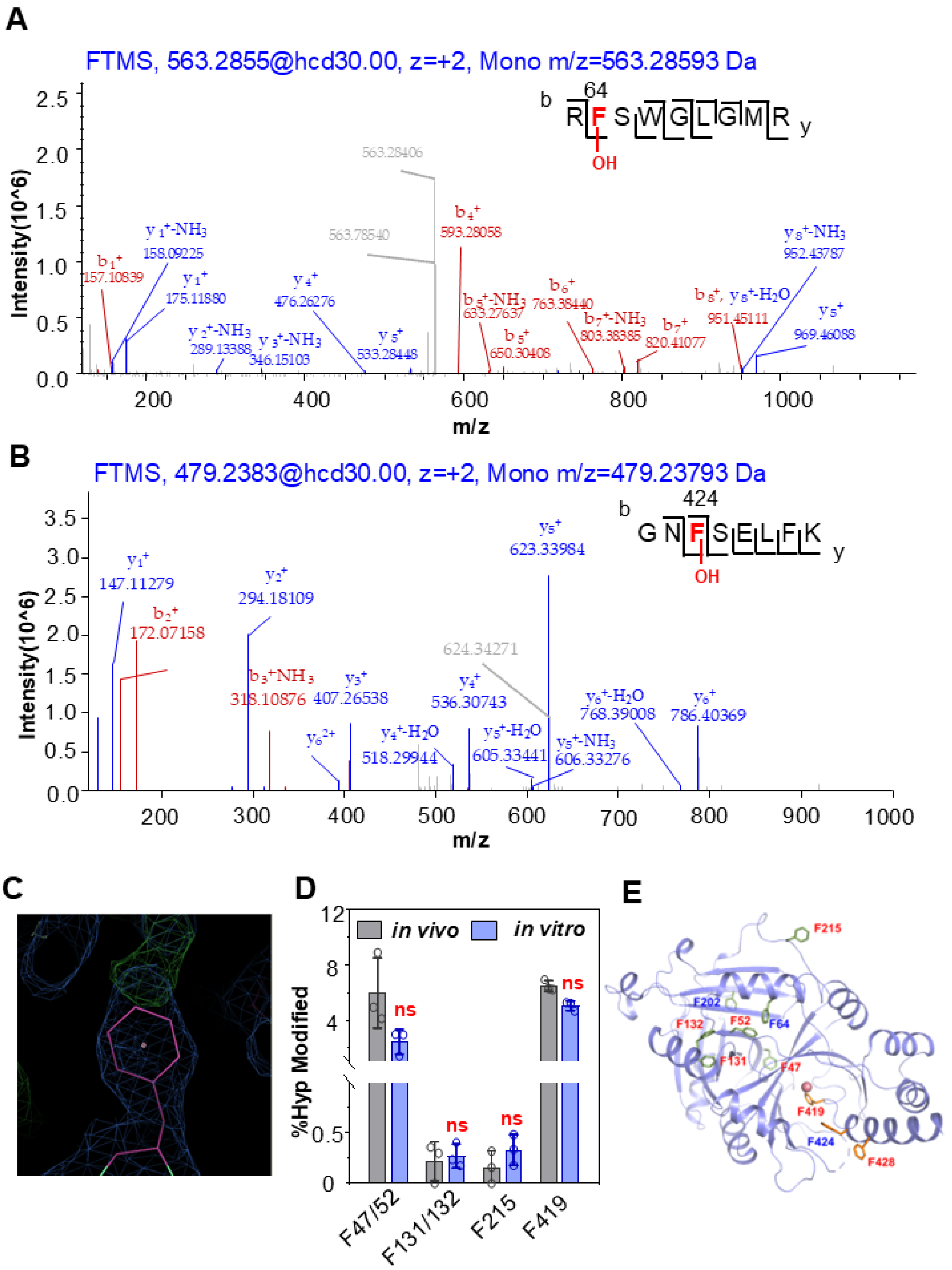

Fig. S3. Identification of HPPD modification sites *in vitro* by LC-MS/MS and crystal structures. (A) The product ion spectrum of the residue F64 of *At*HPPD. (B) The product ion spectrum of the residue F424 of *At*HPPD. (C) Density map of F132 sidechain in the *At*HPPD crystal structures showed the modification of hydroxylation. (D) Comparison of hydroxylation levels *in vitro* and *in vivo*. ns means not significant. %Hyp Modified refers to the level of hydroxylation. (E) Hydroxylation sites identified by LC-MS/MS. Red indicates mofied sites in both *in vivo* and *in vitro*;

blue indicates modified sites only *in vitro*.

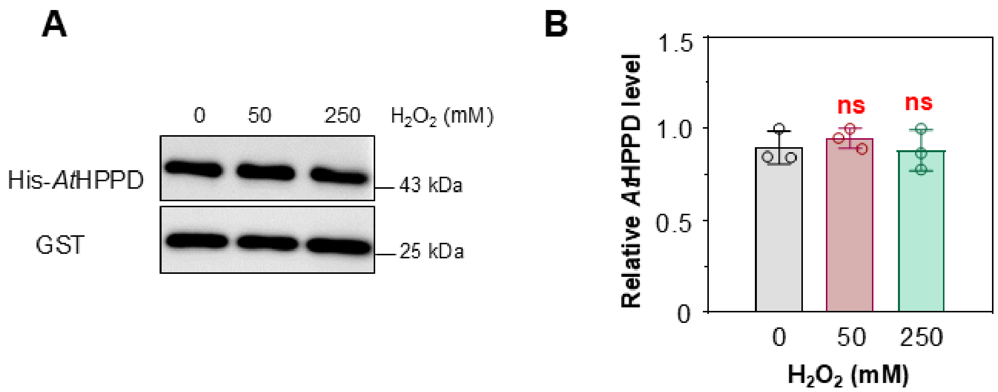

Fig. S4. H_2O_2 cannot directly degrade *At*HPPD. (A) The degradation of *At*HPPD is not directly induced in the presence of 50 mM and 250 mM H_2O_2. GST served as a loading control. (B) Relative band densities of the His-*At*HPPD protein shown in (A), as quantified using Touch view with 0 h set to 1. Data are means of three replicates, and the individual results for each replicate are shown. Significant differences compared with 0 h were determined using Student's t-test: ns means not significant.

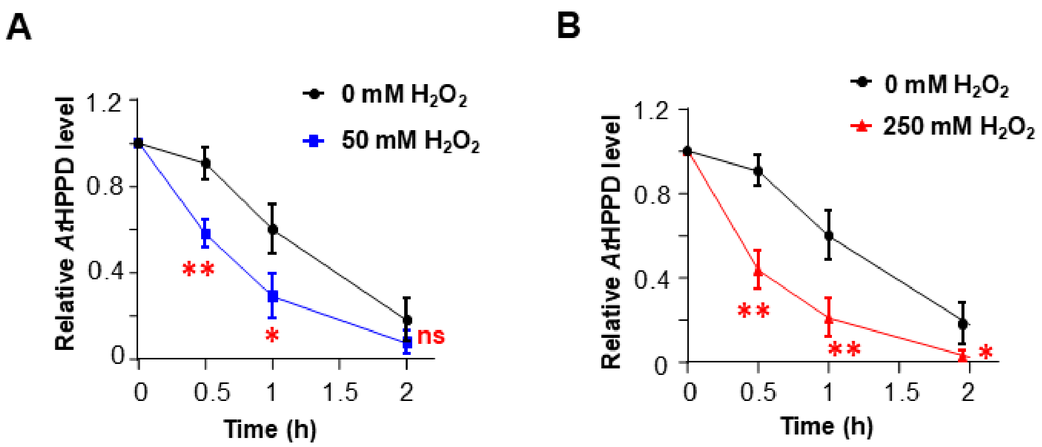

Fig. S5. The effect of H_2O_2 on the stability of *At*HPPD in an *in vitro* cell-free degradation assay. (A) Dose-response curves of the relative *At*HPPD levels at 0 mM and 50 mM H_2O_2 shown in (Fig. 2A). (B) Dose-response curves of the relative *At*HPPD levels at 0 mM and 250 mM H_2O_2 shown in (Fig. 2A). Data are means of three replicates, and the individual results for each replicate are shown. * means $P < 0.05$, ** means $P < 0.01$, ns means not significant.

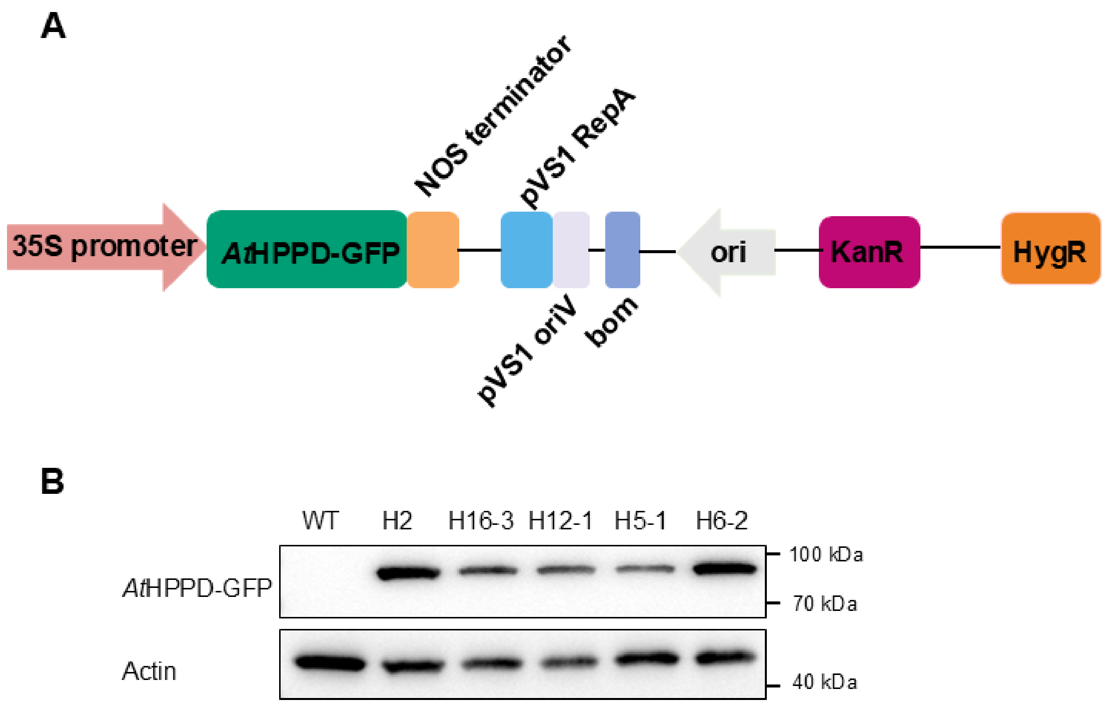

Fig. S6. Expression level of transgenic lines expressing *At*HPPD-GFP. (A) The pBWA(V)HS vector for expression of *At*HPPD in *Arabidopsis thaliana*. KanR and HygR, kanamycin and hygromycin resistance genes. (B) Expression profiles determined by immunoblotting analysis. Actin served as a loading control.

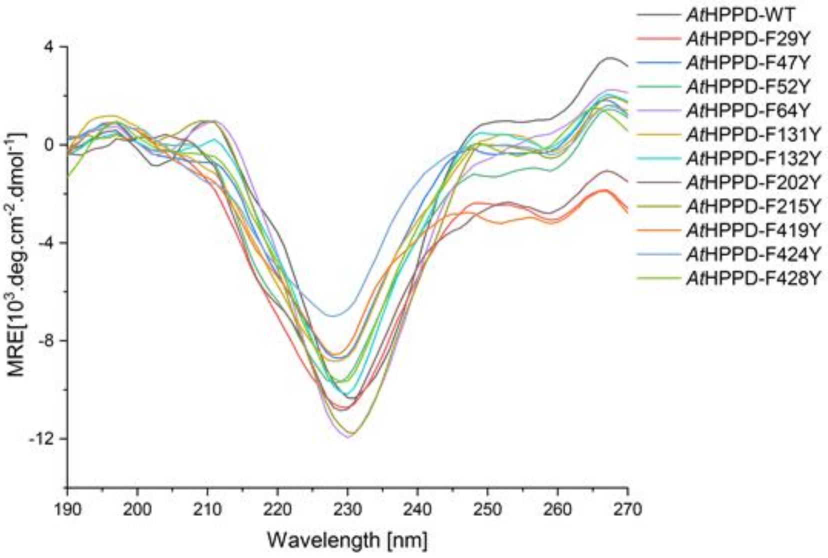

Fig. S7. CD spectra of *At*HPPD and its mutants.

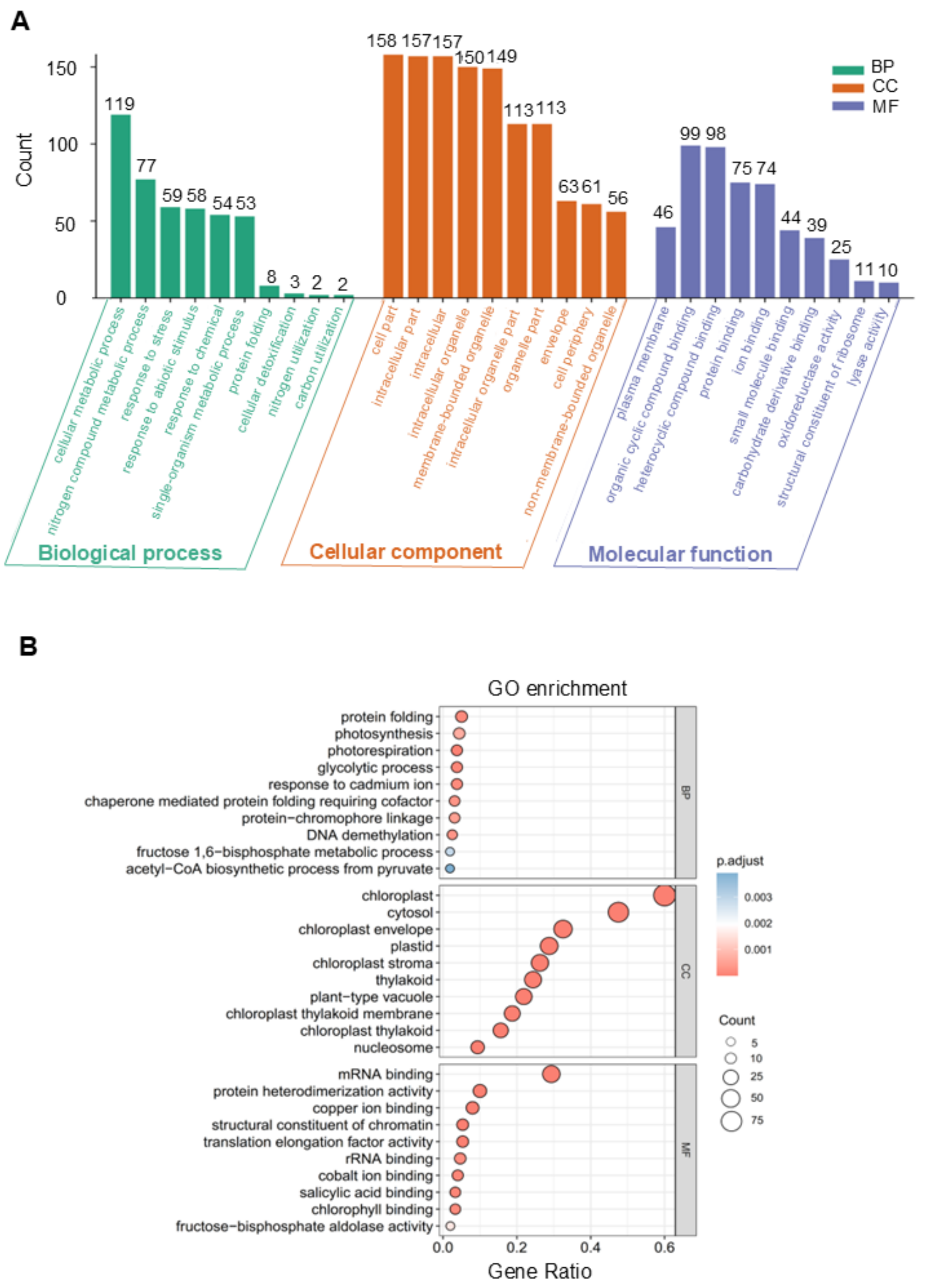

Fig. S8. GO classification and enrichment analysis of the identified the interaction proteins of *At*HPPD. (A) Functional annotation of the interaction proteins of *At*HPPD based on biological process, cellular component and molecular function, which were significantly in accordance with hyper p value which is < 0.05. BP means biological process, CC means cellular component, MF means molecular function. (B) GO enrichment analysis of the interaction proteins of *At*HPPD. A higher gene ratio indicates a greater level of enrichment. The size of the circles in the figure represents the number of proteins associated with each GO category, with larger circles indicating a higher number of associated proteins.

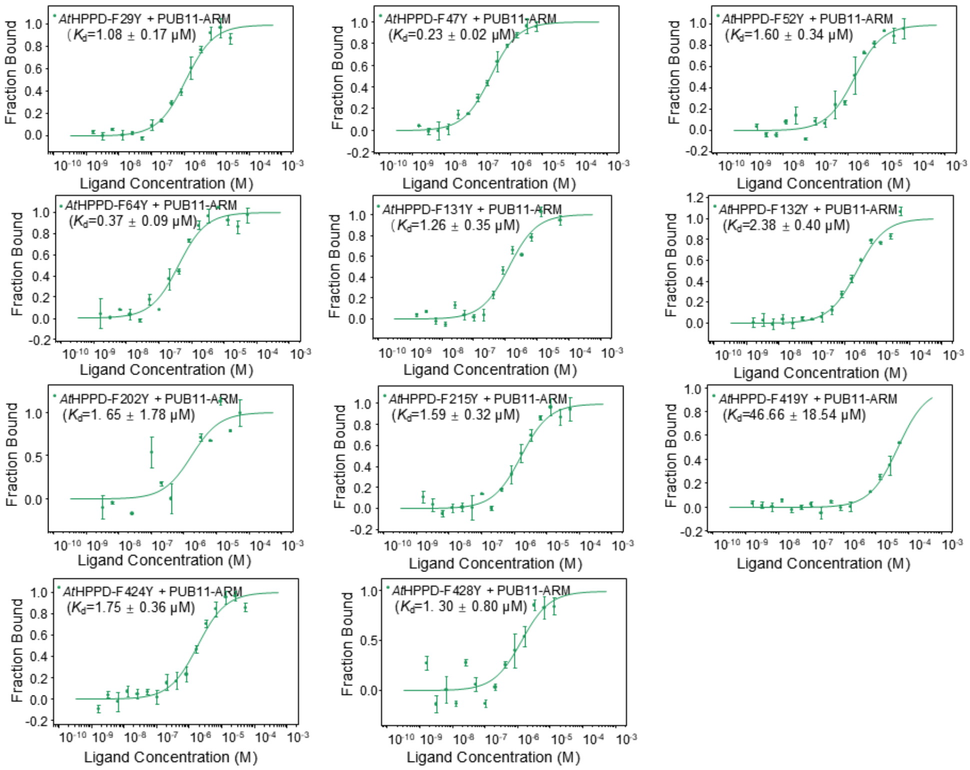

Fig. S9. MST-based interaction analysis between hydroxylated *At*HPPD mutants and PUB11.

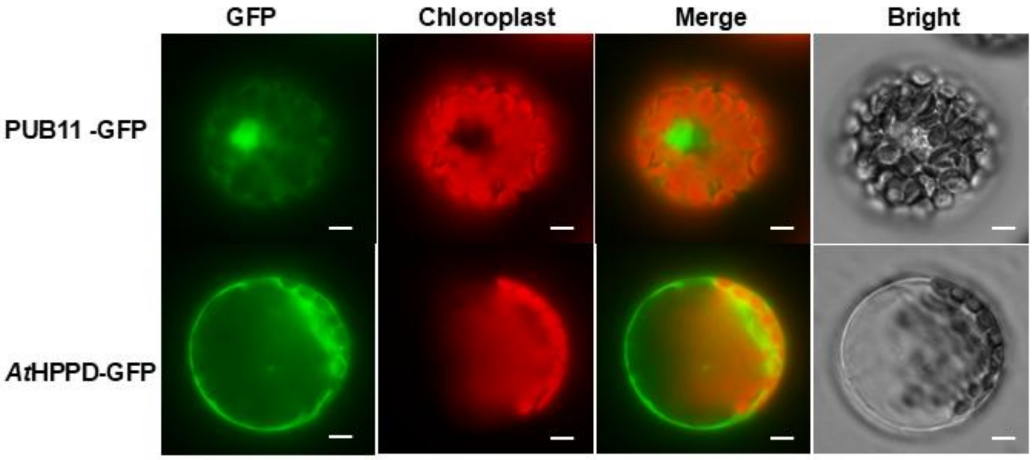

Fig. S10. Subcellular localization of PUB11 and *At*HPPD. PUB11-GFP and *At*HPPD-GFP constructs were respectively transformed into *Arabidopsis protoplasts*. Bars = 10 μm.

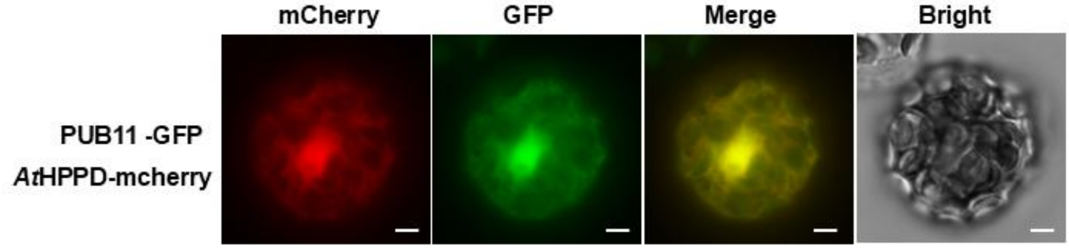

Fig. S11. Co-localization of PUB11 and *At*HPPD. PUB11-GFP and *At*HPPD-mcherry constructs were co-transformed into *Arabidopsis* protoplasts. Bars = 10 µm.

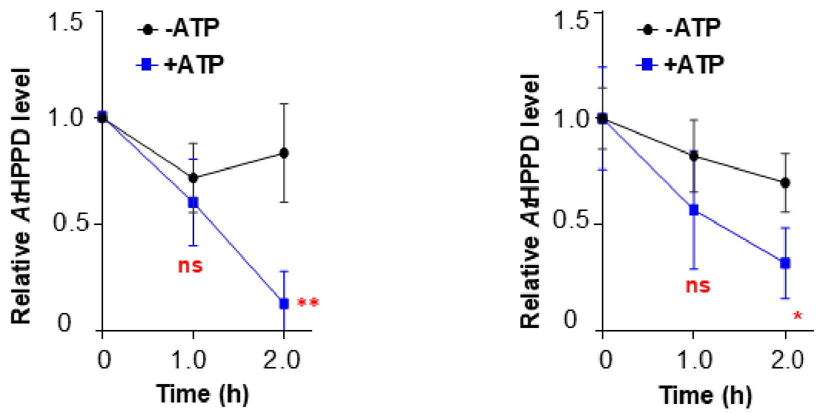

Fig. S12. ATP-dependent enhancement of *At*HPPD degradation. Dose–response curves of relative band intensity of the *At*HPPD-GFP protein in *At*HPPD-GFP-OE (H2 and H6-2) plants shown in (Fig. 4A), as quantified using Touch view with 0 h set to 1 Data are means of three replicates, and the individual results for each replicate are shown. The abundance of *At*HPPD at the 0 min (-ATP, +ATP) was set to 1, respectively. Significant differences compared with 0 h were determined using Student’s t-test: * means P < 0.05, ** means P < 0.01, ns means not significant.

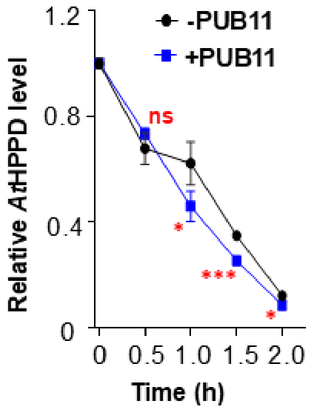

Fig. S13. PUB11 enhances *At*HPPD degradation *in vitro*. Dose–response curves of relative band intensity of the *At*HPPD protein shown in (Fig.4D), as quantified using touch view with 0 h set to 1. Data are means of three replicates, and the individual results for each replicate are shown. Significant differences compared with 0 h were determined using Student's t-test: * means $P < 0.05$, *** means $P < 0.001$, ns means not significant.

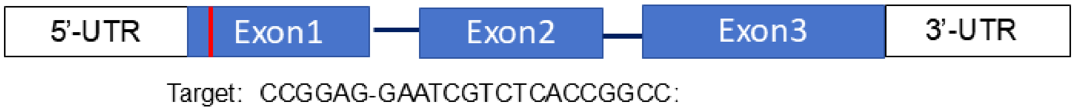

WT: GCGACGGCGTAATGGCCGGAGGAATCGTCTCACCGGCCTCTCTA
pub11(#1): GCGACGGCGTAATGGCCGGAGTGAATCGTCTCACCGGCCTCTCTA (insert 1bp)
pub11(#2): GCGACGGCGTAATGGCCGGAG-GAATCGTCTCACCGGCCTCTCTA (deletion 1bp)

Fig. S14. Identification of *pub11* for the creation of CRISPR/Cas9 knockout lines.

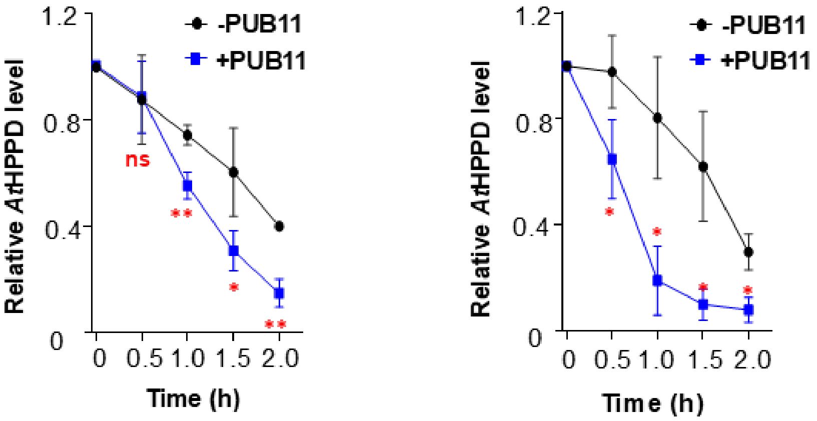

Fig. S15. Cell-free degradation of His-*At*HPPD in *pub11* extracts with or without recombinant PUB11 protein. Dose–response curves of relative band intensity of the *At*HPPD protein shown in (Fig.4E), as quantified using touch view with 0 h set to 1. Data are means of three replicates, and the individual results for each replicate are shown. Significant differences compared with 0 h were determined using Student's t-test: * means $P < 0.05$, ** means $P < 0.01$, ns means not significant.

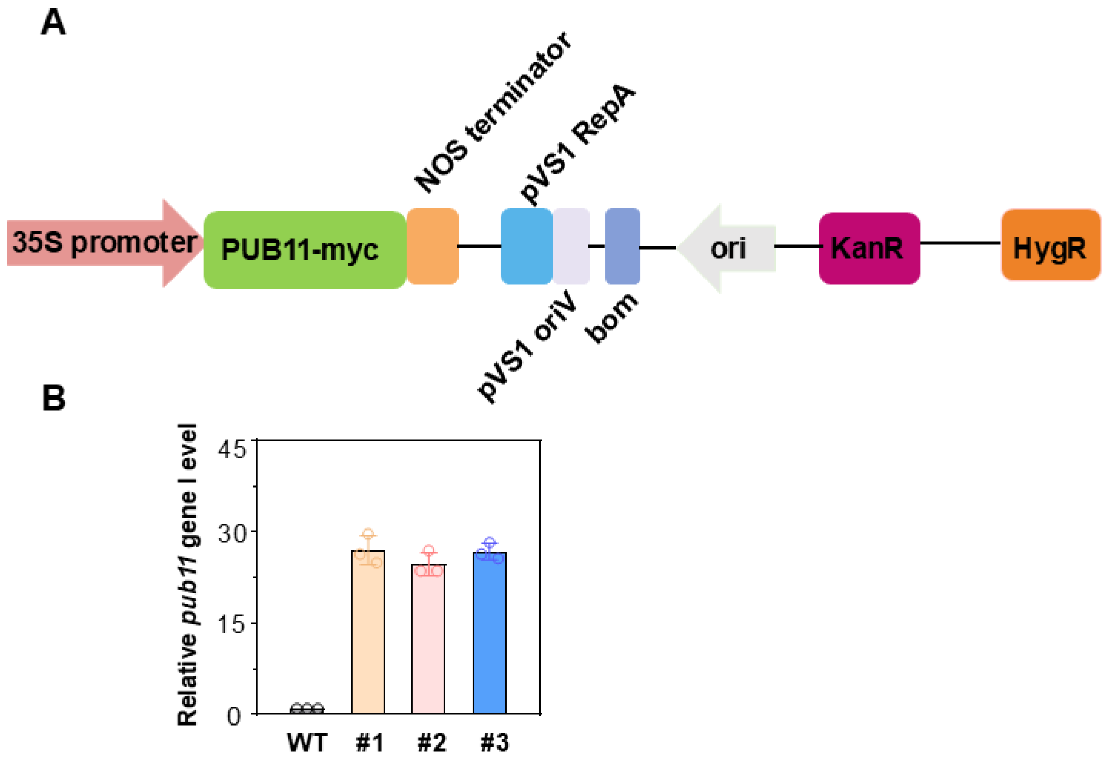

Fig. S16. Expression level of transgenic lines and wild-type plants. (A). The pCAMBIA 3300 vector for expression of PUB11-OE in *Arabidopsis thaliana*. KanR and HygR, kanamycin and hygromycin resistance genes. (B). *Arabidopsis thaliana* RT-PCR results for transgenic gene. WT, wild type plants; #1~#3, PUB11 overexpressing plants.

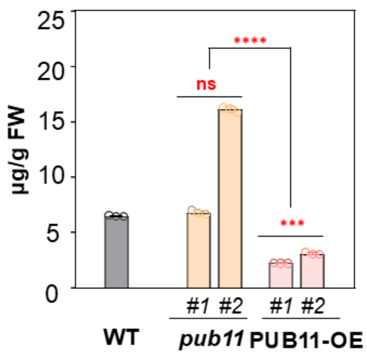

Fig. S17. Tocopherol content determination. *** means P < 0.001, **** means P < 0.0001, ns means not significant. Error bars indicate ±s.d.

Table S1 *At*HPPD mutants exhibited no detectable hydroxylation modifications

Mutants	Annotated sequence	RT (min)	Hydroxylation sequence
*At*HPPD-F29A	G**A**SKFVR	38.3912	Not identified
*At*HPPD-F47A	R**A**HHIEFWCGDATNVATNVA	32.5861	Not identified
*At*HPPD-F52A	FHHIE**A**WCGDATNVATNVA	34.1440	Not identified
*At*HPPD-F64A	R**A**SWGLGMR	20.9285	Not identified
*At*HPPD-F131A	S**A**FSSHGLGVR	20.1026	Not identified
*At*HPPD-F132A	SF**A**SSHGLGVR	15.8525	Not identified
*At*HPPD-F202A	AEDTEKSE**A**LPGFER	21.2350	Not identified
*At*HPPD-F215A	VEDASS**A**PLDYGIR	31.4586	Not identified
*At*HPPD-F419A	AYQSGGCGG**A**GK	8.3512	Not identified
*At*HPPD-F424A	GN**A**SELFK	37.0387	Not identified
*At*HPPD-F428A	KGNFSEL**A**K	12.8025	Not identified

Table S2 Comparison of apparent kinetic parameters for reaction of the *At*HPPD with H_2O_2. Each experiment was carried out in triplicate.

The concentration of H_2O_2(mM)	K_m(μM)	k_{cat}(s^{-1})	k_{cat}/K_m($s^{-1}μM^{-1}$)
0	2.061 ± 0.069	0.200 ± 0.010	0.097
50	2.699 ± 0.071	0.070 ± 0.004	0.026
250	12.354 ± 0.610	0.076 ± 0.001	0.006

Table S4 Oligonucleotide primers used in the study

Primer	Sequence（5' to 3'）
GFP-*At*HPPD-F	GAGCATATACGCCCGGAGTC
GFP-*At*HPPD-R	CAAGACCTGCCTGAAACCGA
pub11-F	TGCGATACCTGCCTTAGTT
pub11-R	GGCTCTGACTGCTCTACCTTT
actin-F	TCCCGCTATGTATGTCGC
actin-R	GCTGGTCTTTGAGGTTTCC
